# Crystal structure of benzyl *N*′-[(1*E*,4*E*)-1,5-bis­(4-meth­oxy­phen­yl)penta-1,4-dien-3-yl­idene]hydrazine-1-carbodi­thio­ate

**DOI:** 10.1107/S2056989019013458

**Published:** 2019-10-03

**Authors:** Nabeel Arif Tawfeeq, Huey Chong Kwong, Mohamed Ibrahim Mohamed Tahir, Thahira B. S. A. Ravoof

**Affiliations:** a Department of Chemistry, Faculty of Science, Universiti Putra Malaysia, 43400 UPM Serdang, Selangor Darul Ehsan, Malaysia; b Department of Chemistry, College of Education for Women, University of Anbar, Iraq

**Keywords:** crystal structure, Schiff base, hydrazinecarbodi­thio­ate bridge, inter­molecular inter­actions, data survey

## Abstract

In the title hydrazinecarbodi­thio­ate derivative, C_27_H_26_N_2_O_2_S_2_, the asymmetric unit is comprised of four mol­ecules (*Z* = 8 and *Z*′ = 4). The 4-meth­oxy­phenyl rings are slightly twisted away from their attached olefinic double bonds [torsion angles = 5.9 (4)–19.6 (4)°]. The azomethine double bond has an *s-trans* configuration relative to one of the C=C bonds and an *s-cis* configuration relative to the other [C=C—C= N = 147.4 (6)–175.7 (2) and 15.3 (3)–37.4 (7)°, respectively]. The torsion angles between the azomethine C=N double bond and hydrazine-1-carbodi­thio­ate moiety indicate only small deviations from planarity, with torsion angles ranging from 0.9 (3) to 6.9 (3)° and from 174.9 (3) to 179.7 (2)°, respectively. The benzyl ring and the methyl­enesulfanyl moiety are almost perpendicular to each other, as indicated by their torsion angles [range 93.7 (3)–114.6 (2)°]. In the crystal, mol­ecules are linked by C—H⋯O, N—H⋯S and C—H⋯π(ring) hydrogen-bonding inter­actions into a three-dimensional network. Structural details of related benzyl hydrazine-1-carbodi­thio­ate are surveyed and compared with those of the title compound.

## Abstract 2

The title compound comprises four crystallographically different mol­ecules that are composed of a 1,5-bis­(4-meth­oxy­phen­yl)penta-1,4-dien-3-ylidenyl group and a benzyl ring connected by a hydrazine-1-carbodi­thio­ate bridge. In the crystal, mol­ecules are connected into a three-dimensional network through C—H⋯O, N—H⋯S and C—H⋯π inter­actions.

## Chemical context   

*S*-benzyl and *S*-alkyl di­thio­carbaza­tes are inter­esting ligands in coordination chemistry because they can act as *N,S*-chelating agents because of the presence of soft sulfur and hard nitro­gen donor atoms (Takjoo *et al.*, 2016 ▸). These types of ligands can form stable metal complexes with five-membered chelate rings, and with transition metals in different stable oxidation states (Centore *et al.*, 2013 ▸). Di­thio­carbazate Schiff bases and their metal complexes show a wide range of biological activities such as anti-malarial, anti-bacterial, anti-viral and anti-tumour (Low *et al.*, 2016 ▸; Nanjundan *et al.*, 2016 ▸; Islam *et al.*, 2016*a* ▸). *S*-benzyl­dithio­carbazate and *S*-alkyl­dithio­carbazate Schiff base derivatives formed with aromatic aldehydes and ketones as well as their metal complexes have attracted attention due to their cytotoxicity against many types of cancer cell lines (Yusof *et al.*, 2016 ▸; 2017*a* ▸,*b* ▸; Vijayan *et al.*, 2015 ▸; Basha *et al.*, 2012 ▸), whereby *S*-methyl and *S*-benzyl di­thio­carbazate Schiff bases with 2-acetyl­pyridine show better cytotoxicity against a breast cancer cell line (MDA-MB-231) than their transition-metal complexes (Hamid *et al.*, 2016 ▸). Furthermore, Schiff bases synthesized from the reaction of *S*-benzyl­dithio­carbazate and *m*-hy­droxy­aceto­phenone as well as their metal complexes exhibit moderate analgesic and good anti-inflammatory activities in comparison with standard drugs diclofenac sodium and indomethacin (Mahapatra *et al.*, 2017 ▸).
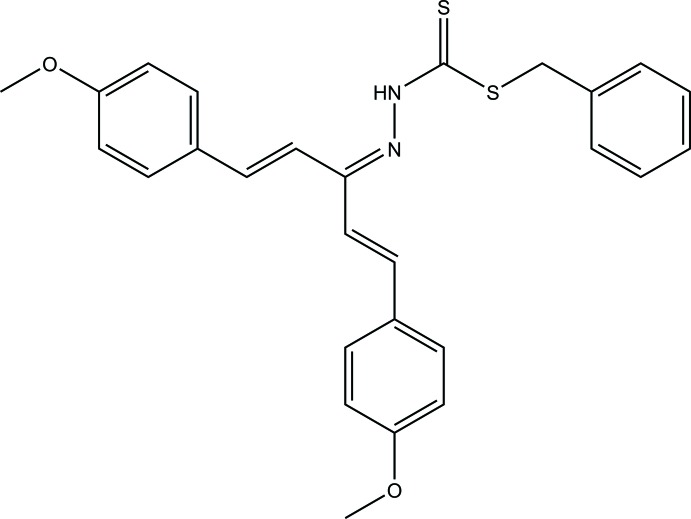


Encouraged by previous findings on various properties of related Schiff base derivatives, we report herein the synthesis and structure determination of the title compound (**I**). Structural details of (**I**) are compared with other hydrazinecarbodi­thio­ates.

## Structural commentary   

**Figure 1:**
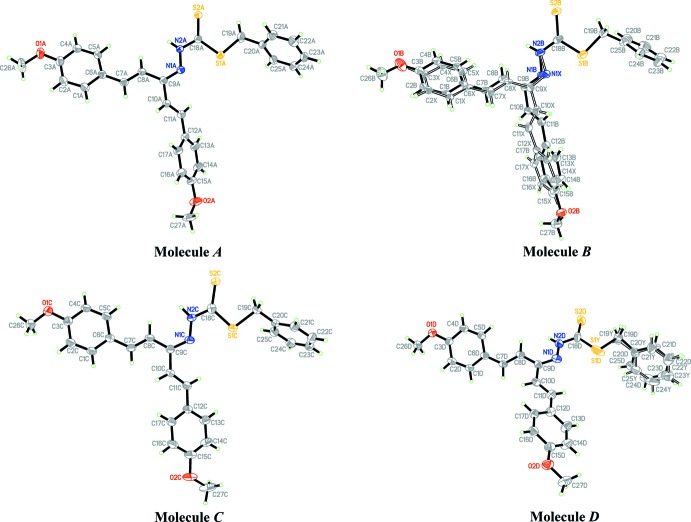
The structures of the four independent mol­ecules in (**I**), showing 50% probability displacement ellipsoids and the atomic labelling scheme.

**Figure 2:**
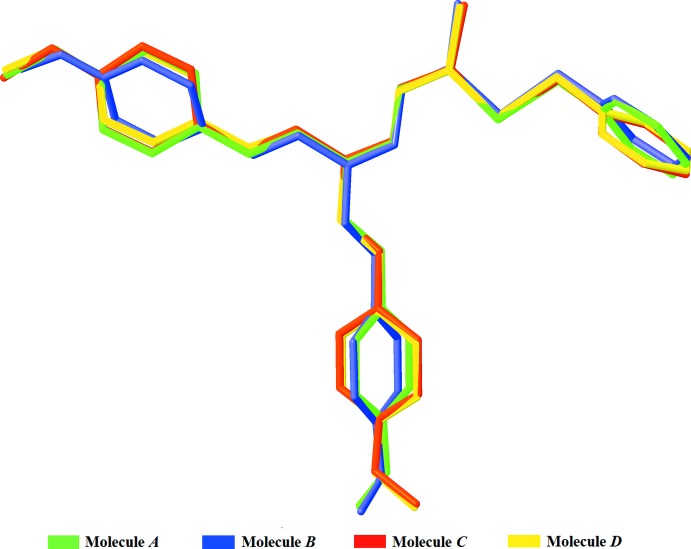
Overlay of the four mol­ecules in (**I**). The r.m.s deviation of *A:B* = 1.038 Å, *A:C* = 0.881 Å and *A:D* = 0.947 Å.

The asymmetric unit of (**I**) consists of four mol­ecules, denoted as *A*, *B*, *C* and *D*. Orientational disorder of the 1,5-bis(phen­yl)penta-1,4-dien-3-ylidenyl amine moiety in mol­ecule *B* and of the phenyl­methyl moiety in mol­ecule *D* were observed. The mol­ecules are composed of a 1,5-bis­(4-meth­oxy­phen­yl)penta-1,4-dien-3-ylidenyl moiety, connected to a benzyl ring by a hydrazine-1-carbodi­thio­ate (–C=N—(NH)—(C=S)—S—C–) bridge (Fig. 1 ▸). Conformational details of the 1,5-bis­(4-meth­oxy­phen­yl)penta-1,4-dien-3-ylidenyl moiety can be described by the torsion angles between the 4-meth­oxy moiety and the phenyl ring [*τ*_1_ (C2—C3—O1—C26); *τ*_6_ (C16—C15—O2—C27)], between the 4-meth­oxy­phenyl ring and the olefinic double bond [*τ*_2_ (C5—C6-C7—C8); *τ*_5_ (C17—C12—C11—C10)], and between the olefinic double bond and the azomethine double bond [*τ*_3_ (N1—C9—C8—C7); *τ*_4_ (N1—C9—C10—C11)]. Torsion angles *τ*_1_ and *τ*_6_ are approximately 0° or ±180° in the majority of the four mol­ecules, except mol­ecule *C* which has a *τ*_1_ of 20.9 (3)° (Table 1 ▸). The 4-meth­oxy­phenyl rings in the 1,5-bis­(4-meth­oxy­phen­yl)penta-1,4-dien-3-ylidenyl moiety are twisted away from their attached olefinic double bonds [*τ*_2_ = 5.9 (4)–16.4 (13)°; *τ*_5_ = 7.5 (3)–19.6 (4)°]. The orientations of the the azomethine double bond with its neighbouring olefinic double bonds relative to the inter­mediate C—C bond (C8—C9; C9—C10) are different: one is in *s-trans* [*τ*_3_ = 147.4 (6)–175.7 (2)°] conformation and the other in *s-cis* [*τ*_4_ = 15.3 (3)–37.4 (7)°] conformation. The dihedral angles between two 4-meth­oxy­phenyl rings in an individual mol­ecule are in the range 23.59 (12)–89.6 (5)° (Table 2 ▸). Conformational details of the benzyl hydrazine-1-carbodi­thio­ate moiety can be outlined by torsion angles *τ*_7_ (C9—N1—N2—C18), *τ*_8_ (N1—N2—C18—S1), *τ*_9_ (N2—C18—S1—C19), *τ*_10_ (C18—S1—C19—C20) and *τ*_11_ (S1—C19—C20—C21). In all mol­ecules of (**I**), the hydrazine-1-carbo­thio­ate bridges are more or less planar (ideal values *τ*_7_, *τ*_8_ and *τ*_9_ = 0 or ±180°; experimental values: 0.9 (3)–6.9 (3)° and 174.9 (3)–179.7 (2)°, respectively). The torsion angles between the sulfane moiety and the methyl­ene moiety indicate a slight twist [*τ*_10_ = 160.53 (17)–163.36 (16)°]. These contortions are more severe between the benzyl ring and the methyl­ene sulfane moiety where *τ*_11_ is considerably smaller [*τ*_11_ = 93.7 (3)–114.6 (2)°]. The dihedral angles between the benzyl ring and the two 4-meth­oxy­phenyl rings are in the range 31.6 (5)–89.9 (8)° (Table 2 ▸). An overlay of the four mol­ecules in the asymmetric unit was created with *OLEX2* (Dolomanov *et al.*, 2009 ▸) and is shown in Fig. 2 ▸.

## Supra­molecular features   

**Figure 3:**
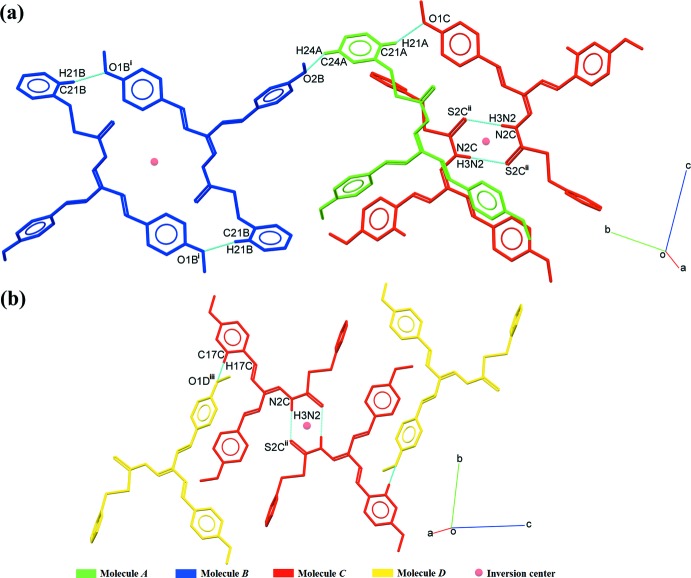
A partial packing diagram of the title compound, with N—H⋯S and C—H⋯O inter­actions (dotted lines). Hydrogen atoms not involved in these inter­actions were omitted for clarity.

**Figure 4:**
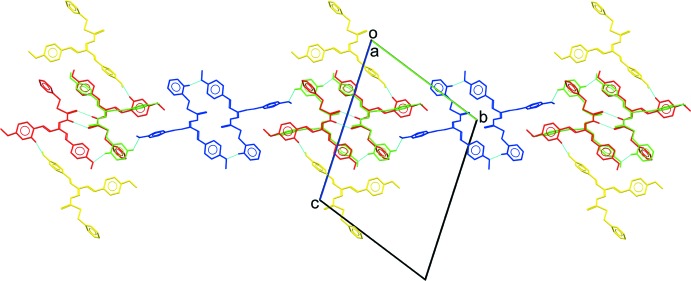
A partial packing diagram of the title compound showing mol­ecules linked into chains by N—H⋯S and C—H⋯O inter­actions.

**Figure 5:**
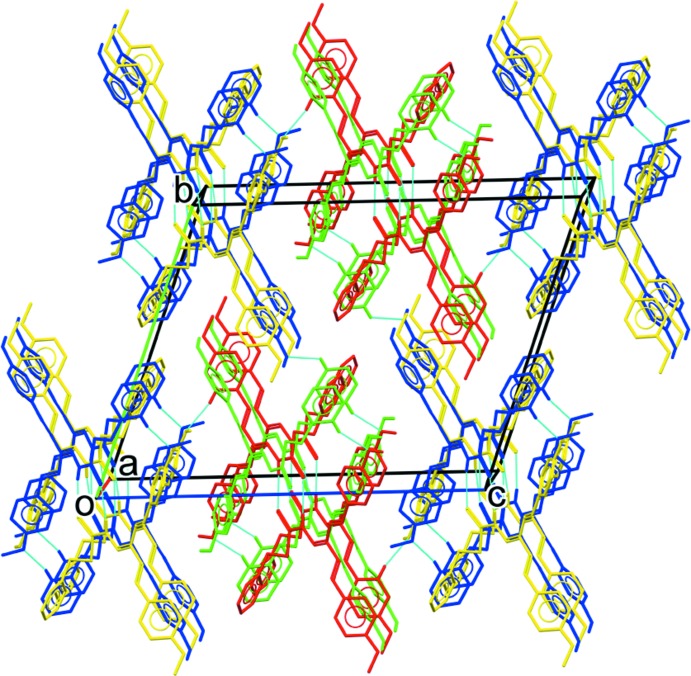
The overall packing of the title compound, viewed approximately along the *a-*axis direction.

In the crystal, mol­ecule *A* is inter­connected to mol­ecule *B* and mol­ecule *C* through weak C24*A*—H24*A*⋯O2*B* and C21*A*—H21*A*⋯O1*C* hydrogen-bonding inter­actions. Mol­ecules *B* and *C* each form inversion-related dimers *via* C21*B*—H21*B*⋯O1*B* and N2*C*—H3*N*2⋯S2*C* inter­actions, respectively (Fig. 3 ▸ *a*). In addition, mol­ecule *C* and mol­ecule *D* are connected through C17*C*—H17*C*⋯O1*D* hydrogen bonds (Fig. 3 ▸ *b*). The four mol­ecules are linked into an endless chain parallel to [021] through the combination of these hydrogen bonds (Fig. 4 ▸). Further inter­actions, namely N2*A*—H1*N*2⋯S2*A*, N2*B*—H2*N*2⋯S2*D* and N2*D*—H4*N*2⋯S2*B*, link the chains into a three-dimensional network, as shown in Fig. 5 ▸. Additional C—H⋯π inter­actions (Table 3 ▸) consolidate the packing.

## Database survey   

**Figure 6:**
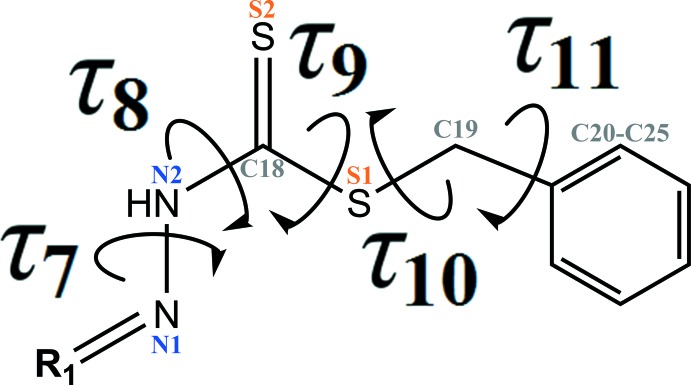
General chemical scheme showing the definition of torsion angles, *τ*_7_, *τ*_8_, *τ*_9_, *τ*_10_ and *τ*_11_ in the benzyl hydrazine-1-carbo­thio­ate moiety.

A search of the Cambridge Structural Database (CSD, version 5.40, last update May 2019; Groom *et al.*, 2016 ▸) using benzyl 2-(λ^2^-methyl­ene)hydrazine-1-carbodi­thio­ate as reference moiety resulted in 45 structures with different substituents. The reference moiety and relevant torsion angles are illus­trated in Fig. 6 ▸. Details regarding different substituents (***R_1_***) together with the torsion angles for the benzyl hydrazine-1-carbo­thio­ate moiety in these structures are collated in Table 4 ▸. In analogy with the title mol­ecules, the planarity of the hydrazine-1-carbodi­thio­ate bridge for these structures is indicated by the *τ*_7_, *τ*_8_ and *τ*_9;_ torsion angles *τ*_7_ and *τ*_9_ range from 165.1 to 180.0° and indicate an *anti*-periplanar conformation whereas torsion angle *τ*_8_ is indicative of a *syn*-periplanar conformation (0.0– 9.1°). With respect to torsion angle *τ*_10_, most of the structures adopt an *anti*-periplanar conformation ranging from 159.5 to 180.0°, but there are nine structures that adopt either a *syn*-clinal or an *anti*-clinal conformation (77.6–110.4°). In most of the structures, the benzyl ring and the methyl­ene sulfane moiety are orientated almost perpendicular to each other, as indicated by torsion angle *τ*_11_. Here, either a *syn*-clinal (68.1–88.4°) or an *anti*-clinal (92.2–141.1°) conformation is adopted. However, there is one outlier (WADGAK; Chan *et al.*, 2003 ▸) where the benzyl hydrazine-1-carbodi­thio­ate moiety is substituted with a 1-(thio­phen-2-yl)ethyl­idenyl moiety. In contrast to most of the structures, torsion angle *τ*_11_ = 17.1° for WADGAK indicates a *syn*-periplanar conformation.

## Synthesis and crystallization   

**Figure 7:**
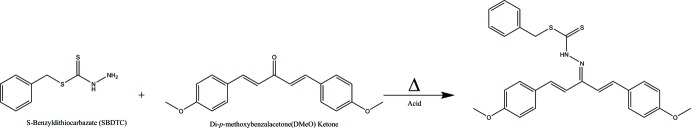
Reaction scheme for the synthesis of (**I**).

Compound (**I**) was synthesized following a well-described literature protocol (Ali & Tarafder, 1977 ▸; Ravoof *et al.*, 2010 ▸; Omar *et al.*, 2014 ▸). *S*-benzyl­dithio­carbazate (1.98 g, 0.01 mol) was dissolved in absolute ethanol (50 ml) under heating and stirring. The resulting solution was slowly added to a hot solution of di-*p*-meth­oxy­benzalacetone (2.94 g, 0.01 mol) dissolved in absolute ethanol (50 ml). 3-5 drops of concentrated hydro­chloric acid were added to the mixture, which was subsequently heated and stirred for 5 h (Fig. 7 ▸). The acidified mixture was allowed to stand overnight, resulting in the formation of red crystals. They were filtered off and recrystallized using the slow evaporation technique from absolute ethanol as solvent.

Yield: 57.3%. m.p.: 375-376 K. Analysis calculated for C_27_H_26_N_2_O_2_S_2_: C, 49.71; H, 5.52; N, 5.90; S, 13.51% found: C, 49.68; H, 5.48; N, 5.97; S, 13.55%. FT-IR (cm^−1^): 3145, ν(N—H); 1630, ν(C=N); 1244, ν(N—N); 1059, ν(C=S); 3145. ^1^H NMR (CDCl_3_) δ (p.p.m.)= 6.55–7.68 (aromatic H); 3.85 (OCH_3_); 4.55 (–CH_2_ benz­yl); 10.20 (N—H). ^13^C NMR (CDCl_3_) δ(p.p.m.)= 114.35–142.88 (aromatic C); 55.55 (OCH_3_); 39.34 (–CH_2_ benz­yl); 161.22 (C=N); 206.91 (C=S). *m*/*z* calculated for C_27_H_26_N_2_O_2_S_2_: 474, found 474.

## Refinement   

Crystal data, data collection and structure refinement details are summarized in Table 5 ▸. The labelling of atoms is the same in each mol­ecule, with the mol­ecule indicated by the suffix *A*, *B*, *C* or *D*. The N-bound H atoms were located in difference-Fourier maps and were refined freely [N—H = 0.81 (3)–0.91 (4) Å]. The C-bound H atoms were positioned geometrically (C—H = 0.93–0.97 Å) and refined using a riding model, with *U*_iso_(H) = 1.2 or 1.5*U*_eq_(C). A rotating-group model was applied to the methyl groups. The 1,5-bis­(phen­yl)penta-1,4-dien-3-ylidenyl amine moiety in mol­ecule *B* and the phenyl­methyl moiety in mol­ecule *D* display positional disorder, with refined site occupancy ratios of 0.667 (7):0.333 (7) and 0.653 (15):0.347 (15), respectively (SIMU, DELU and SAME restraints were used).

## Supplementary Material

Crystal structure: contains datablock(s) I. DOI: 10.1107/S2056989019013458/wm5520sup1.cif

Structure factors: contains datablock(s) I. DOI: 10.1107/S2056989019013458/wm5520Isup2.hkl

Supporting information file. DOI: 10.1107/S2056989019013458/wm5520Isup3.cml

CCDC references: 1902915, 1902915

Additional supporting information:  crystallographic information; 3D view; checkCIF report

## Figures and Tables

**Table 1:** Selected torsion angles (°) in the four mol­ecules of (**I**) Torsion angles for the minor disorder component were omitted.

Mol­ecule	τ	*A*	*B*	*C*	*D*
C2—C3—O1—C26	*τ*_1_	−2.8 (4)	−7.3 (15)	20.9 (3)	5.0 (4)
C9—N1—N2—C18	*τ*_7_	−178.1 (2)	−175.6 (4)	−179.3 (2)	179.7 (2)
C5—C6—C7—C8	*τ*_2_	5.9 (4)	16.4 (13)	15.9 (4)	−9.3 (4)
N1—N2—C18—S1	*τ*_8_	175.76 (16)	−0.9 (3)	6.9 (3)	2.6 (3)
N1—C9—C8—C7	*τ*_3_	175.0 (2)	147.4 (6)	−175.7 (2)	−156.8 (2)
N2—C18—S1—C19	*τ*_9_	179.43 (17)	−178.15 (17)	−176.64 (17)	−174.9 (3)
N1—C9—C10—C11	*τ*_4_	−37.5 (3)	−37.4 (7)	−26.9 (3)	−15.3 (3)
S1—C19—C20—C21	*τ*_10_	160.53 (17)	−160.75 (17)	−163.36 (16)	−162.9 (6)
C17—C12—C11—C10	*τ*_5_	19.6 (4)	−18.4 (7)	7.5 (3)	15.9 (3)
S1—C19—C20—C21	*τ*_11_	−97.0 (2)	93.7 (3)	−114.6 (2)	−108.6 (12)
C16—C15—O2—C27	*τ*_6_	−0.9 (4)	4.1 (16)	177.5 (3)	177.9 (2)

**Table 2:** Selected dihedral angles (°) Dihedral angle 1 is the dihedral angle between the mean planes of the 4-meth­oxy­phenyl rings; dihedral angle 2 is the dihedral angle between the mean planes of the benzyl (C20–C25) and 4-meth­oxy­phenyl (C1–C6) rings; dihedral angle 3 is the dihedral angle between the mean planes of the benzyl (C20–C25) and the 4-meth­oxy­phenyl (C12–C17) rings.

Mol­ecule	Dihedral angle 1	Dihedral angle 2	Dihedral angle 3
*A*	56.72 (11)	85.02 (11)	72.11 (2)
*B*	89.6 (5)	31.6 (5)	64.9 (3)
*C*	23.59 (12)	84.46 (12)	86.00 (13)
*D*	60.82 (12)	36.6 (8)	89.9 (8)

**Table 3:** Hydrogen-bond geometry (Å, °) *Cg*1, *Cg*2 and *Cg*3 are the centroids of the C12*B*–C17*B*, C12*C*–C17*C* and C1*B*–C6*B* rings, respectively.

*D*—H⋯*A*	*D*—H	H⋯*A*	*D*⋯*A*	*D*—H⋯*A*
C24*A*—H24*A*⋯O2*B*	0.93	2.57	3.262 (3)	131
C21*A*—H21*A*⋯O1*C*	0.93	2.40	3.249 (3)	152
C21*B*—H21*B*⋯O1*B* ^i^	0.93	2.33	3.240 (4)	167
N2*C*—H3*N*2⋯S2*C* ^ii^	0.91 (4)	2.53 (4)	3.435 (2)	179 (3)
C17*C*—H17*C*⋯O1*D* ^iii^	0.93	2.53	3.381 (3)	152
N2*A*—H1*N*2⋯S2*A* ^iv^	0.81 (3)	2.69 (3)	3.473 (2)	161 (2)
N2*B*—H2*N*2⋯S2*D* ^v^	0.82 (3)	2.65 (3)	3.461 (2)	171 (3)
N2*D*—H4*N*2⋯S2*B* ^v^	0.91 (4)	2.61 (4)	3.517 (2)	174 (3)
C14*C*—H14*C*⋯*Cg*1^iv^	0.93	2.80	3.558 (5)	139
C16*A*—H16*A*⋯*Cg*2^ii^	0.93	2.80	3.680 (2)	158
C2*D*—H2*DA*⋯*Cg*3	0.93	2.94	3.700 (5)	140

Symmetry codes: (i) 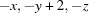; (ii) 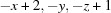; (iii) 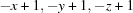; (iv) 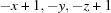; (v) 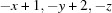.

**Table 4:** Torsion angles *τ*_7_, *τ*_8_, *τ*_9_, *τ*_10_ and *τ*_11_ (°) in related hydrazinecarbodi­thio­ates Two sets of torsion angles are stated for compounds EXINAB, QORJAK, SIMMUX, VOJGUX and WUPGIX because there are two mol­ecules in their asymmetric units. The mol­ecule with disorder in the structure of compound ZENLIN was omitted from this table.

Compound	****R*_1_***	*τ*_7_	*τ*_8_	*τ*_9_	*τ*_10_	*τ*_11_
ABOROA (Manan *et al.*, 2011 ▸)	5-fluoro-2-oxo-1,2-di­hydro-3*H*-indol-3-yliden­yl	175.1	5.0	−176.8	168.2	−98.3
ABORUG (Manan *et al.*, 2011 ▸)	5-bromo-2-oxo-1,2-di­hydro-3*H*-indol-3-yliden­yl	−178.5	−9.1	176.9	−108.0	−84.1
ABOSAN (Manan *et al.*, 2011 ▸)	5-chloro-2-oxo-1,2-di­hydro-3*H*-indol-3-yliden­yl	−178.8	−7.7	177.5	−110.4	−81.8
BAHWIT (Md Yusof *et al.*, 2015 ▸)	2-meth­oxy­benzyl­iden­yl	−179.1	2.6	−178.0	−177.7	−85.7
BAHWOZ (Md Yusof *et al.*, 2015 ▸)	3-meth­oxy­benzyl­iden­yl	−178.0	−1.2	−178.1	173.8	−80.1
CEFBIB (Islam *et al.*, 2016*a* ▸)	2,4,5-tri­meth­oxy­benzyl­iden­yl	179.6	−5.2	−177.9	173.4	−79.7
COBQUH (Mirza *et al.*, 2014 ▸)	pyridin-2-yl­methylen­yl	−177.2	−5.3	−177.2	−176.0	−68.7
DATFEK (Khoo *et al.*, 2005 ▸)	furan-2-yl)ethyl­iden­yl	178.6	1.7	180.0	180.0	−72.5
EDETUD (How *et al.*, 2007 ▸)	1-(3-pyrid­yl)ethyl­iden­yl	−179.2	4.9	−179.8	−165.8	−87.5
EHIXUQ (Yusof *et al.*, 2016 ▸)	2-hy­droxy-3-meth­oxy­benzyl­iden­yl	177.4	1.8	179.9	−173.3	−101.2
EMEBAA (Ravoof *et al.*, 2011 ▸)	bis­(pyridin-2-yl)methylen­yl	−165.7	1.4	175.4	176.2	−83.4
EPOFAR (Ali *et al.*, 2011 ▸)	2-oxo-1,2-di­hydro-3*H*-indol-3-yliden­yl	176.5	−4.5	−176.2	165.0	−95.5
EVITUZ (Shan *et al.*, 2011*a* ▸)	2-nitro­benzyl­iden­yl	170.6	3.6	179.4	−80.1	132.1
EXINAB (Shan *et al.*, 2011*b* ▸)	2-methyl­benzyl­iden­yl	−174.0 −176.9	−2.7 −4.9	−178.7 −177.4	174.2 175.6	−68.2 −68.7
GUMJUV (Break *et al.*, 2013 ▸)	4-chloro­phen­yl)ethyl­iden­yl	−172.7	−0.3	−179.3	86.8	68.1
HELZIK (Omar *et al.*, 2018 ▸)	1-(6-methyl­pyridin-2-yl)ethyl­iden­yl	−177.7	−1.6	−176.0	171.6	−108.0
IFUTUZ (Khaledi *et al.*, 2008*a* ▸)	1*H*-indol-3-yl­methyl­iden­yl	−172.7	2.6	179.0	103.7	−68.2
JAMMOA (Ali *et al.*, 2004 ▸)	quinolinyl-2-methylen­yl	177.9	0.8	177.5	−171.0	101.8
KAGZOK (Ravoof *et al.*, 2015 ▸)	1-(5-methyl­pyridin-2-yl)ethyl­iden­yl	−176.9	−5.6	−173.2	164.7	−77.8
KUCRAC (Hamid *et al.*, 2009 ▸)	1-(pyrazin-2-yl)ethyl­iden­yl	178.4	0.6	178.8	174.7	−70.0
KUCZUD (Xu *et al.*, 1991 ▸)	pyridine-*N*-oxide-2-yl)methyl­iden­yl	−171.4	−2.0	176.5	−161.7	−92.2
LOBSEB (Shan *et al.*, 2008*b* ▸)	furan-2-yl­methylen­yl	173.8	−0.5	179.7	177.1	−93.0
LOBVOO (Tarafder *et al.*, 2008 ▸)	(*E*)-3-phenyl­allyl­iden­yl	−177.4	3.0	177.6	−175.3	−102.7
LOJBUI (Khaledi *et al.*, 2008*b* ▸)	1-methyl-1*H*-indol-2-yl­methylen­yl	179.1	−2.3	−179.1	172.9	−78.9
LOJLIG (Khaledi *et al.*, 2008*b* ▸)	di­phenyl­methylen­yl	170.5	−1.2	−175.2	164.8	−83.8
LUBNIH (Zangrando *et al.*, 2015 ▸)	1-(3-hy­droxy­phen­yl)ethyl­iden­yl	−177.5	−0.8	173.7	180.0	−71.1
NIZBUV (Omar *et al.*, 2014 ▸)	1-(4-methyl­pyridin-2-yl)ethyl­iden­yl	−178.4	0.0	−175.8	−95.9	−141.1
OQOWOH (Akbar Ali *et al.*, 2011 ▸)	bis­(pyridin-2-yl)methylen­yl	165.1	1.9	−177.2	−180.0	−83.8
PEWLEL (Li *et al.*, 2012 ▸)	phen­yl(pyridin-2-yl)methylen­yl	−177.0	4.6	−173.1	87.6	−119.2
QORJAK (Tan *et al.*, 2015 ▸)	3,4-di­meth­oxy­benzyl­iden­yl	169.2 −170.4	−3.1 3.7	−179.5 175.1	169.5 −168.8	−118.8 −105.7
QUCLIL (Biswal *et al.*, 2015 ▸)	1-(2-hy­droxy­phen­yl)ethyl­idene2-hy­droxy­phen­yl)ethyl­iden­yl	176.7	0.6	−173.3	171.2	−115.5
RIYZOP (Shan *et al.*, 2008*a* ▸)	benzyl­iden­yl	177.1	0.1	178.5	171.8	−80.0
RUGLAH (Li *et al.*, 2009 ▸)	3-nitro­benzyl­iden­yl	178.3	−2.9	−177.5	173.1	−85.5
SALSEE (Zhang *et al.*, 2004 ▸)	4-((2-hy­droxy­eth­yl)methyl­amino)­benzyl­iden­yl	−166.4	0.9	−176.8	170.4	−88.4
SIMMUX (Qiu & Luo, 2007 ▸)	2-bromo­benzyl­iden­yl	175.6 −173.9	2.6 −5.6	178.8 −176.3	−170.6 173.6	−115.6 75.2
TADVEC (Islam *et al.*, 2016*b* ▸)	3,4,5-tri­meth­oxy­benzyl­iden­yl	−179.4	−5.1	−177.1	174.9	−73.3
TIFSEH (Roy *et al.*, 2007 ▸)	2-methyl­thio-6-methyl-4-pyrimid­yl)methylen­yl	−177.8	0.6	−178.4	164.9	−76.6
UWATOD (Flörke & Boshaala, 2016 ▸)	1-phenyl­ethyl­iden­yl	172.4	−4.0	−173.3	159.5	−102.0
VOJCUT (Khaledi *et al.*, 2008*c* ▸)	1-methyl-1*H*-indol-3-yl­methyl­iden­yl	−177.2	−1.7	−176.0	−101.3	−129.4
VOJGUX (Shi *et al.*, 2008 ▸)	2-chloro­benzyl­iden­yl	−174.7 178.3	−2.0 0.3	−179.3 176.9	−174.0 −174.1	−80.3 −110.1
WADGAK (Chan *et al.*, 2003 ▸)	1-(thio­phen-2-yl)ethyl­iden­yl	174.8	−5.8	177.8	77.6	17.1
WUPGIX (Tarafder *et al.*, 2002 ▸)	5-methyl­furan-2-yl)methylen­yl	−169.2 179.8	−0.2 1.5	179.7 −176.3	−173.6 −171.7	−100.8 −77.9
YAHDAO (Fan *et al.*, 2011*a* ▸)	4-meth­oxy­benzyl­iden­yl	−175.8	1.4	−179.5	−178.9	−87.9
YAHDUI (Fan *et al.*, 2011*b* ▸)	2-bromo-5-meth­oxy­benzyl­iden­yl	177.8	−3.6	−176.0	174.9	119.2
ZENLIN (Fun *et al.*, 1995 ▸)	4-(di­methyl­amino)­benzyl­iden­yl	−180.0	1.3	179.9	−178.0	−97.1

**Table 5:** Experimental details

Crystal data	
Chemical formula	C_27_H_26_N_2_O_2_S_2_
*M*_r_	474.62
Crystal system, space group	Triclinic, *P* 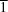
Temperature (K)	100
*a*, *b*, *c* (Å)	10.2010 (1), 20.0260 (3), 25.2349 (4)
α, β, γ (°)	70.535 (1), 86.852 (1), 82.147 (1)
*V* (Å^3^)	4814.65 (12)
*Z*	8
Radiation type	Cu *K*α
μ (mm^−1^)	2.22
Crystal size (mm)	0.14 × 0.08 × 0.05

Data collection	
Diffractometer	XtaLAB Synergy, Dualflex, AtlasS2
Absorption correction	Gaussian (*CrysAlis PRO*; Rigaku OD, 2018 ▸)
*T*_min_, *T*_max_	0.681, 1.000
No. of measured, independent and observed [*I* > 2σ(*I*)] reflections	114750, 17182, 14177
*R*_int_	0.046
(sin θ/λ)_max_ (Å^−1^)	0.597

Refinement	
*R*[*F* ^2^ > 2σ(*F* ^2^)], *wR*(*F* ^2^), *S*	0.048, 0.130, 1.03
No. of reflections	17182
No. of parameters	1449
No. of restraints	80
H-atom treatment	H atoms treated by a mixture of independent and constrained refinement
Δρ_max_, Δρ_min_ (e Å^−3^)	1.16, −0.62

Computer programs: *CrysAlis PRO* (Rigaku OD, 2018 ▸), *SHELXS97* (Sheldrick, 2008 ▸), *SHELXL2013* (Sheldrick, 2015 ▸), *Mercury* (Macrae *et al.*, 2006 ▸), *OLEX2* (Dolomanov *et al.*, 2009 ▸) and *PLATON* (Spek, 2009 ▸).

## supplementary crystallographic information

### Crystal data

**Table:**

C_27_H_26_N_2_O_2_S_2_	*Z* = 8
*M**_r_* = 474.62	*F*(000) = 2000
Triclinic, *P*1	*D*_x_ = 1.310 Mg m^−^^3^
*a* = 10.2010 (1) Å	Cu *K*α radiation, λ = 1.54178 Å
*b* = 20.0260 (3) Å	Cell parameters from 39902 reflections
*c* = 25.2349 (4) Å	θ = 3.7–76.4°
α = 70.535 (1)°	µ = 2.22 mm^−^^1^
β = 86.852 (1)°	*T* = 100 K
γ = 82.147 (1)°	Plate, clear intense red
*V* = 4814.65 (12) Å^3^	0.14 × 0.08 × 0.05 mm

### Data collection

**Table:**

XtaLAB Synergy, Dualflex, AtlasS2 diffractometer	17182 independent reflections
Radiation source: micro-focus sealed X-ray tube, PhotonJet (Cu) X-ray Source	14177 reflections with *I* > 2σ(*I*)
Detector resolution: 5.2558 pixels mm^-1^	*R*_int_ = 0.046
ω scans	θ_max_ = 67.1°, θ_min_ = 3.5°
Absorption correction: gaussian (CrysAlis PRO; Rigaku OD, 2018)	*h* = −12→12
*T*_min_ = 0.681, *T*_max_ = 1.000	*k* = −23→22
114750 measured reflections	*l* = −30→30

### Refinement

**Table:**

Refinement on *F*^2^	80 restraints
Least-squares matrix: full	Hydrogen site location: mixed
*R*[*F*^2^ > 2σ(*F*^2^)] = 0.048	H atoms treated by a mixture of independent and constrained refinement
*wR*(*F*^2^) = 0.130	*w* = 1/[σ^2^(*F*_o_^2^) + (0.0653*P*)^2^ + 3.3132*P*] where *P* = (*F*_o_^2^ + 2*F*_c_^2^)/3
*S* = 1.03	(Δ/σ)_max_ = 0.001
17182 reflections	Δρ_max_ = 1.16 e Å^−^^3^
1449 parameters	Δρ_min_ = −0.62 e Å^−^^3^

### Special details

**Table:**

Geometry. All esds (except the esd in the dihedral angle between two l.s. planes) are estimated using the full covariance matrix. The cell esds are taken into account individually in the estimation of esds in distances, angles and torsion angles; correlations between esds in cell parameters are only used when they are defined by crystal symmetry. An approximate (isotropic) treatment of cell esds is used for estimating esds involving l.s. planes.

### Fractional atomic coordinates and isotropic or equivalent isotropic displacement parameters (Å^2^)

**Table:**

	*x*	*y*	*z*	*U*_iso_*/*U*_eq_	Occ. (<1)
S1A	0.38286 (5)	0.22017 (3)	0.45386 (2)	0.02996 (13)	
S2A	0.33363 (5)	0.06508 (3)	0.50610 (2)	0.02843 (12)	
N1A	0.59006 (18)	0.16709 (9)	0.40187 (8)	0.0289 (4)	
N2A	0.52876 (18)	0.11192 (10)	0.43731 (8)	0.0273 (4)	
H1N2	0.553 (3)	0.0696 (15)	0.4436 (11)	0.035 (7)*	
O1A	1.10882 (18)	−0.19587 (9)	0.33832 (8)	0.0455 (4)	
O2A	0.78919 (19)	0.52640 (9)	0.13693 (8)	0.0502 (5)	
C1A	1.0517 (2)	−0.00371 (12)	0.31844 (10)	0.0327 (5)	
H1AA	1.090814	0.037742	0.301784	0.039*	
C2A	1.1166 (2)	−0.06786 (13)	0.31486 (11)	0.0360 (5)	
H2AA	1.197486	−0.069235	0.296124	0.043*	
C3A	1.0584 (2)	−0.12966 (12)	0.33974 (10)	0.0344 (5)	
C4A	0.9379 (2)	−0.12664 (12)	0.36779 (10)	0.0354 (5)	
H4AA	0.899450	−0.168273	0.384749	0.043*	
C5A	0.8748 (2)	−0.06274 (12)	0.37073 (10)	0.0315 (5)	
H5AA	0.793795	−0.061597	0.389444	0.038*	
C6A	0.9311 (2)	0.00086 (11)	0.34583 (9)	0.0275 (4)	
C7A	0.8665 (2)	0.07035 (11)	0.34688 (9)	0.0283 (4)	
H7AA	0.907514	0.109891	0.326213	0.034*	
C8A	0.7556 (2)	0.08318 (11)	0.37416 (9)	0.0288 (4)	
H8AA	0.714877	0.044069	0.395916	0.035*	
C9A	0.6929 (2)	0.15396 (11)	0.37257 (9)	0.0281 (4)	
C10A	0.7424 (2)	0.21836 (11)	0.33310 (9)	0.0299 (5)	
H10A	0.833252	0.218739	0.327660	0.036*	
C11A	0.6612 (2)	0.27617 (11)	0.30489 (10)	0.0326 (5)	
H11A	0.571218	0.274950	0.312663	0.039*	
C12A	0.6996 (2)	0.34134 (11)	0.26292 (10)	0.0320 (5)	
C13A	0.6064 (2)	0.38630 (12)	0.22410 (11)	0.0375 (5)	
H13A	0.520152	0.375220	0.226469	0.045*	
C14A	0.6390 (3)	0.44668 (12)	0.18239 (11)	0.0404 (6)	
H14A	0.575534	0.475249	0.156663	0.048*	
C15A	0.7669 (2)	0.46478 (12)	0.17885 (11)	0.0379 (5)	
C16A	0.8617 (2)	0.42146 (12)	0.21703 (11)	0.0375 (5)	
H16A	0.947445	0.433161	0.214852	0.045*	
C17A	0.8274 (2)	0.36054 (11)	0.25847 (10)	0.0337 (5)	
H17A	0.891163	0.331773	0.283925	0.040*	
C18A	0.4203 (2)	0.12795 (11)	0.46564 (9)	0.0264 (4)	
C19A	0.2365 (2)	0.22242 (13)	0.49776 (13)	0.0448 (7)	
H19A	0.159869	0.217668	0.478795	0.054*	
H19B	0.247377	0.182943	0.532824	0.054*	
C20A	0.2159 (2)	0.29220 (11)	0.50951 (10)	0.0309 (5)	
C21A	0.2611 (2)	0.29552 (13)	0.55912 (11)	0.0399 (6)	
H21A	0.307545	0.255074	0.584080	0.048*	
C22A	0.2387 (3)	0.35777 (14)	0.57229 (11)	0.0419 (6)	
H22A	0.268796	0.358855	0.606080	0.050*	
C23A	0.1716 (2)	0.41825 (12)	0.53528 (11)	0.0371 (5)	
H23A	0.156331	0.460290	0.543993	0.044*	
C24A	0.1273 (2)	0.41627 (12)	0.48555 (11)	0.0387 (5)	
H24A	0.082566	0.457230	0.460470	0.046*	
C25A	0.1486 (2)	0.35363 (12)	0.47231 (10)	0.0342 (5)	
H25A	0.117832	0.352740	0.438563	0.041*	
C26A	1.2328 (3)	−0.20308 (17)	0.31144 (17)	0.0622 (9)	
H26A	1.257882	−0.252198	0.314525	0.093*	
H26B	1.298221	−0.187579	0.329079	0.093*	
H26C	1.226313	−0.174351	0.272485	0.093*	
C27A	0.9199 (3)	0.54696 (15)	0.13175 (14)	0.0591 (8)	
H27A	0.923718	0.589567	0.100000	0.089*	
H27B	0.982668	0.509279	0.126530	0.089*	
H27C	0.940797	0.555781	0.165270	0.089*	
S1B	0.39730 (6)	0.76943 (3)	0.04835 (2)	0.03568 (14)	
S2B	0.43703 (5)	0.92506 (3)	−0.00786 (2)	0.03000 (13)	
N2B	0.2514 (2)	0.87613 (11)	0.06618 (9)	0.0382 (5)	
H2N2	0.227 (3)	0.9186 (15)	0.0605 (12)	0.035 (7)*	
O1B	−0.31580 (19)	1.19059 (12)	0.15074 (8)	0.0544 (5)	
O2B	−0.05549 (17)	0.47568 (8)	0.37333 (7)	0.0382 (4)	
C18B	0.3579 (2)	0.86140 (11)	0.03607 (9)	0.0309 (5)	
C19B	0.5367 (2)	0.76729 (12)	0.00149 (13)	0.0442 (6)	
H19C	0.521337	0.805729	−0.033959	0.053*	
H19D	0.616236	0.773125	0.017978	0.053*	
C20B	0.5526 (2)	0.69606 (12)	−0.00798 (11)	0.0359 (5)	
C21B	0.4943 (3)	0.68917 (15)	−0.05345 (12)	0.0488 (6)	
H21B	0.442817	0.728294	−0.077543	0.059*	
C22B	0.5111 (3)	0.62506 (18)	−0.06389 (13)	0.0573 (8)	
H22B	0.471649	0.621575	−0.095064	0.069*	
C23B	0.5855 (3)	0.56663 (15)	−0.02866 (13)	0.0524 (7)	
H23B	0.598908	0.523844	−0.036292	0.063*	
C24B	0.6400 (3)	0.57196 (13)	0.01807 (12)	0.0516 (7)	
H24B	0.688022	0.532036	0.042923	0.062*	
C25B	0.6243 (3)	0.63638 (13)	0.02859 (12)	0.0440 (6)	
H25B	0.661956	0.639434	0.060301	0.053*	
C26B	−0.3399 (3)	1.21002 (18)	0.20007 (13)	0.0585 (8)	
H26D	−0.383061	1.258173	0.190106	0.088*	
H26E	−0.257378	1.206576	0.217922	0.088*	
H26F	−0.395555	1.178403	0.225470	0.088*	
C27B	−0.1821 (3)	0.45199 (14)	0.37586 (12)	0.0464 (6)	
H27D	−0.185034	0.408726	0.407067	0.070*	
H27E	−0.196837	0.443262	0.341629	0.070*	
H27F	−0.249514	0.488044	0.380591	0.070*	
S1C	1.11763 (6)	−0.22107 (3)	0.54167 (2)	0.03300 (13)	
S2C	1.13700 (7)	−0.06552 (3)	0.47861 (3)	0.04438 (16)	
N1C	0.92728 (19)	−0.16897 (9)	0.60427 (8)	0.0310 (4)	
N2C	0.96835 (19)	−0.11438 (10)	0.56067 (8)	0.0308 (4)	
H3N2	0.941 (3)	−0.0668 (18)	0.5497 (14)	0.062 (9)*	
O1C	0.38467 (17)	0.18373 (9)	0.67618 (8)	0.0424 (4)	
O2C	0.7281 (2)	−0.53080 (10)	0.86542 (9)	0.0595 (5)	
C1C	0.4978 (2)	−0.00762 (13)	0.71071 (10)	0.0383 (5)	
H1CA	0.489065	−0.050567	0.739130	0.046*	
C2C	0.4318 (2)	0.05467 (13)	0.71624 (10)	0.0375 (5)	
H2CA	0.380330	0.053488	0.748040	0.045*	
C3C	0.4429 (2)	0.11881 (12)	0.67410 (10)	0.0324 (5)	
C4C	0.5178 (2)	0.11903 (13)	0.62654 (11)	0.0402 (6)	
H4CA	0.523531	0.161930	0.597630	0.048*	
C5C	0.5838 (2)	0.05681 (12)	0.62143 (10)	0.0377 (5)	
H5CA	0.633646	0.058137	0.589190	0.045*	
C6C	0.5768 (2)	−0.00852 (12)	0.66427 (10)	0.0316 (5)	
C7C	0.6511 (2)	−0.07643 (12)	0.66400 (10)	0.0339 (5)	
H7CA	0.622182	−0.117503	0.689193	0.041*	
C8C	0.7550 (2)	−0.08630 (11)	0.63195 (9)	0.0306 (5)	
H8CA	0.782189	−0.046291	0.604652	0.037*	
C9C	0.8293 (2)	−0.15577 (11)	0.63682 (9)	0.0297 (5)	
C10C	0.7988 (2)	−0.21889 (12)	0.68407 (10)	0.0318 (5)	
H10C	0.760475	−0.211209	0.716178	0.038*	
C11C	0.8219 (2)	−0.28565 (12)	0.68419 (10)	0.0319 (5)	
H11C	0.856297	−0.292752	0.651316	0.038*	
C12C	0.7979 (2)	−0.34955 (12)	0.73162 (10)	0.0321 (5)	
C13C	0.8149 (3)	−0.41598 (12)	0.72411 (11)	0.0399 (6)	
H13C	0.841893	−0.419045	0.689104	0.048*	
C14C	0.7926 (3)	−0.47799 (13)	0.76760 (12)	0.0451 (6)	
H14C	0.803828	−0.521705	0.761452	0.054*	
C15C	0.7540 (3)	−0.47420 (13)	0.81963 (11)	0.0430 (6)	
C16C	0.7396 (2)	−0.40854 (13)	0.82858 (11)	0.0411 (6)	
H16C	0.715610	−0.406011	0.864018	0.049*	
C17C	0.7607 (2)	−0.34747 (12)	0.78525 (10)	0.0343 (5)	
H17C	0.750003	−0.303980	0.791764	0.041*	
C18C	1.0683 (2)	−0.12894 (13)	0.52717 (10)	0.0342 (5)	
C19C	1.2426 (2)	−0.21912 (12)	0.48679 (11)	0.0356 (5)	
H19E	1.211522	−0.184086	0.451463	0.043*	
H19F	1.323596	−0.205913	0.496674	0.043*	
C20C	1.2686 (2)	−0.29216 (11)	0.48084 (9)	0.0304 (5)	
C21C	1.3916 (2)	−0.33277 (13)	0.49416 (12)	0.0403 (6)	
H21C	1.457608	−0.315225	0.507770	0.048*	
C22C	1.4165 (3)	−0.39910 (14)	0.48731 (14)	0.0518 (7)	
H22C	1.498957	−0.425961	0.496461	0.062*	
C23C	1.3198 (3)	−0.42546 (13)	0.46702 (13)	0.0470 (7)	
H23C	1.337010	−0.469916	0.462221	0.056*	
C24C	1.1982 (3)	−0.38611 (14)	0.45391 (12)	0.0437 (6)	
H24C	1.132802	−0.404143	0.440387	0.052*	
C25C	1.1717 (2)	−0.31965 (13)	0.46060 (11)	0.0378 (5)	
H25C	1.088770	−0.293331	0.451523	0.045*	
C26C	0.2749 (2)	0.18433 (14)	0.71364 (12)	0.0421 (6)	
H26G	0.235743	0.232671	0.707503	0.063*	
H26H	0.304629	0.163257	0.751748	0.063*	
H26I	0.210361	0.157478	0.706877	0.063*	
C27C	0.7472 (4)	−0.59989 (16)	0.85860 (16)	0.0765 (11)	
H27G	0.720411	−0.634840	0.892439	0.115*	
H27H	0.838932	−0.612035	0.851000	0.115*	
H27I	0.694871	−0.598980	0.827822	0.115*	
S2D	0.87082 (7)	0.94461 (4)	−0.02823 (3)	0.05084 (18)	
N1D	0.67804 (19)	0.84475 (11)	0.10430 (9)	0.0368 (4)	
N2D	0.71944 (19)	0.89845 (11)	0.06061 (9)	0.0353 (4)	
H4N2	0.680 (3)	0.9447 (19)	0.0493 (15)	0.066 (10)*	
O1D	0.17094 (18)	1.21299 (9)	0.17203 (9)	0.0464 (4)	
O2D	0.4571 (2)	0.49382 (9)	0.36916 (8)	0.0481 (4)	
C1D	0.2135 (2)	1.02822 (12)	0.17183 (11)	0.0360 (5)	
H1DA	0.165625	0.989735	0.179164	0.043*	
C2D	0.1496 (2)	1.09145 (13)	0.17662 (11)	0.0389 (5)	
H2DA	0.060380	1.095586	0.186367	0.047*	
C3D	0.2215 (2)	1.14876 (12)	0.16658 (10)	0.0352 (5)	
C4D	0.3540 (2)	1.14194 (13)	0.15068 (11)	0.0391 (6)	
H4DA	0.401831	1.180437	0.143535	0.047*	
C5D	0.4150 (2)	1.07893 (12)	0.14542 (10)	0.0359 (5)	
H5DA	0.503412	1.075558	0.134304	0.043*	
C6D	0.3464 (2)	1.01957 (12)	0.15651 (9)	0.0308 (5)	
C7D	0.4073 (2)	0.95049 (11)	0.15394 (9)	0.0297 (5)	
H7DA	0.356137	0.913176	0.167502	0.036*	
C8D	0.5280 (2)	0.93413 (12)	0.13443 (9)	0.0311 (5)	
H8DA	0.579507	0.970872	0.118385	0.037*	
C9D	0.5835 (2)	0.86182 (12)	0.13692 (10)	0.0310 (5)	
C10D	0.5399 (2)	0.80078 (12)	0.18230 (10)	0.0333 (5)	
H10D	0.490036	0.810412	0.211485	0.040*	
C11D	0.5664 (2)	0.73254 (12)	0.18488 (10)	0.0358 (5)	
H11D	0.606774	0.723634	0.153544	0.043*	
C12D	0.5375 (2)	0.67071 (12)	0.23253 (10)	0.0363 (5)	
C13D	0.5449 (3)	0.60356 (14)	0.22646 (12)	0.0479 (6)	
H13D	0.567937	0.599002	0.191540	0.058*	
C14D	0.5193 (3)	0.54365 (14)	0.27040 (12)	0.0489 (7)	
H14D	0.524588	0.499738	0.264807	0.059*	
C15D	0.4856 (3)	0.54910 (13)	0.32285 (11)	0.0409 (6)	
C16D	0.4824 (2)	0.61498 (13)	0.33092 (11)	0.0386 (5)	
H16D	0.463280	0.618790	0.366341	0.046*	
C17D	0.5073 (2)	0.67434 (12)	0.28650 (11)	0.0354 (5)	
H17D	0.504116	0.717948	0.292432	0.043*	
C18D	0.8177 (2)	0.88359 (15)	0.02564 (13)	0.0453 (6)	
C26D	0.0331 (3)	1.22342 (15)	0.18465 (15)	0.0539 (7)	
H26J	0.009561	1.270676	0.186504	0.081*	
H26K	0.014110	1.188981	0.220161	0.081*	
H26L	−0.017203	1.217610	0.155763	0.081*	
C27D	0.4637 (3)	0.42460 (14)	0.36333 (13)	0.0554 (7)	
H27J	0.436668	0.391242	0.397991	0.083*	
H27K	0.552803	0.409205	0.354291	0.083*	
H27L	0.405868	0.427030	0.333790	0.083*	
C1B	−0.1596 (13)	1.0124 (6)	0.2057 (4)	0.029 (2)	0.667 (7)
H1BA	−0.139365	0.976700	0.239899	0.035*	0.667 (7)
C2B	−0.2170 (17)	1.0787 (6)	0.2066 (4)	0.030 (2)	0.667 (7)
H2BA	−0.231049	1.088033	0.240317	0.036*	0.667 (7)
C3B	−0.2530 (12)	1.1308 (4)	0.1560 (3)	0.0299 (19)	0.667 (7)
C4B	−0.2285 (12)	1.1158 (4)	0.1058 (3)	0.0291 (17)	0.667 (7)
H4BA	−0.253074	1.150920	0.071809	0.035*	0.667 (7)
C5B	−0.1684 (9)	1.0497 (5)	0.1060 (4)	0.0258 (17)	0.667 (7)
H5BA	−0.153061	1.040639	0.072191	0.031*	0.667 (7)
C6B	−0.1304 (8)	0.9961 (5)	0.1564 (3)	0.0209 (16)	0.667 (7)
C7B	−0.0605 (7)	0.9261 (4)	0.1610 (3)	0.0268 (14)	0.667 (7)
H7BA	−0.060254	0.890582	0.195945	0.032*	0.667 (7)
C8B	0.0037 (6)	0.9091 (4)	0.1185 (2)	0.0276 (12)	0.667 (7)
H8BA	0.002944	0.942997	0.082753	0.033*	0.667 (7)
N1B	0.1817 (5)	0.8225 (2)	0.10207 (19)	0.0291 (10)	0.667 (7)
C9B	0.0749 (6)	0.8374 (3)	0.12805 (19)	0.0269 (10)	0.667 (7)
C10B	0.0237 (4)	0.77673 (17)	0.17134 (15)	0.0293 (10)	0.667 (7)
H10B	−0.067534	0.776853	0.174888	0.035*	0.667 (7)
C11B	0.0995 (4)	0.7220 (2)	0.20555 (17)	0.0310 (10)	0.667 (7)
H11B	0.190427	0.723056	0.201807	0.037*	0.667 (7)
C12B	0.0539 (6)	0.6597 (2)	0.24877 (17)	0.0291 (10)	0.667 (7)
C13B	0.1395 (5)	0.6162 (3)	0.2913 (2)	0.0331 (11)	0.667 (7)
H13B	0.224929	0.627323	0.291609	0.040*	0.667 (7)
C14B	0.1013 (8)	0.5567 (4)	0.3331 (4)	0.0309 (17)	0.667 (7)
H14B	0.159726	0.528900	0.361364	0.037*	0.667 (7)
C15B	−0.0261 (14)	0.5392 (7)	0.3321 (7)	0.0276 (18)	0.667 (7)
C16B	−0.1120 (6)	0.5806 (4)	0.2896 (3)	0.0311 (15)	0.667 (7)
H16B	−0.195961	0.568073	0.288374	0.037*	0.667 (7)
C17B	−0.0728 (6)	0.6407 (3)	0.2489 (2)	0.0304 (10)	0.667 (7)
H17B	−0.132224	0.668952	0.221220	0.036*	0.667 (7)
C1X	−0.139 (3)	0.9949 (10)	0.2158 (6)	0.021 (3)	0.333 (7)
H1XB	−0.111834	0.963916	0.251090	0.025*	0.333 (7)
C2X	−0.212 (4)	1.0607 (11)	0.2108 (9)	0.029 (4)	0.333 (7)
H2XB	−0.233806	1.072327	0.243153	0.035*	0.333 (7)
C3X	−0.253 (2)	1.1094 (8)	0.1593 (8)	0.029 (4)	0.333 (7)
C4X	−0.221 (2)	1.0902 (8)	0.1111 (6)	0.026 (4)	0.333 (7)
H4XB	−0.247986	1.121596	0.075905	0.031*	0.333 (7)
C5X	−0.1507 (19)	1.0247 (8)	0.1159 (7)	0.026 (4)	0.333 (7)
H5XB	−0.131880	1.012595	0.083493	0.032*	0.333 (7)
C6X	−0.1057 (17)	0.9753 (8)	0.1680 (7)	0.023 (4)	0.333 (7)
C7X	−0.0213 (13)	0.9091 (8)	0.1718 (6)	0.030 (3)	0.333 (7)
H7XB	−0.010847	0.875995	0.207777	0.036*	0.333 (7)
C8X	0.0432 (11)	0.8893 (6)	0.1309 (4)	0.022 (2)	0.333 (7)
H8XB	0.029093	0.918740	0.093893	0.027*	0.333 (7)
N1X	0.2384 (10)	0.8148 (5)	0.1119 (4)	0.0275 (19)	0.333 (7)
C9X	0.1350 (11)	0.8235 (4)	0.1415 (4)	0.0262 (19)	0.333 (7)
C10X	0.1172 (8)	0.7579 (5)	0.1892 (3)	0.030 (2)	0.333 (7)
H10E	0.192881	0.727915	0.205173	0.036*	0.333 (7)
C11X	0.0016 (7)	0.7388 (3)	0.2107 (3)	0.032 (2)	0.333 (7)
H11E	−0.073871	0.770751	0.197430	0.039*	0.333 (7)
C12X	−0.0153 (12)	0.6696 (4)	0.2549 (3)	0.0274 (18)	0.333 (7)
C13X	0.0832 (11)	0.6316 (5)	0.2930 (4)	0.031 (2)	0.333 (7)
H13E	0.162299	0.649756	0.293077	0.038*	0.333 (7)
C14X	0.0608 (17)	0.5656 (9)	0.3311 (9)	0.040 (5)	0.333 (7)
H14E	0.126458	0.540056	0.356782	0.048*	0.333 (7)
C15X	−0.055 (3)	0.5364 (16)	0.3323 (16)	0.039 (6)	0.333 (7)
C16X	−0.1558 (12)	0.5768 (7)	0.2966 (7)	0.028 (3)	0.333 (7)
H16E	−0.237504	0.560458	0.298540	0.033*	0.333 (7)
C17X	−0.1335 (12)	0.6423 (5)	0.2579 (4)	0.034 (2)	0.333 (7)
H17E	−0.200591	0.668545	0.233116	0.040*	0.333 (7)
S1D	0.8699 (3)	0.79428 (15)	0.04235 (14)	0.0365 (7)	0.653 (15)
C19D	0.9873 (8)	0.7925 (4)	−0.0153 (4)	0.051 (2)	0.653 (15)
H19I	0.950782	0.825342	−0.050551	0.061*	0.653 (15)
H19J	1.069360	0.808008	−0.008995	0.061*	0.653 (15)
C20D	1.0154 (10)	0.7177 (4)	−0.0193 (5)	0.044 (4)	0.653 (15)
C21D	1.1364 (9)	0.6760 (6)	−0.0038 (8)	0.061 (4)	0.653 (15)
H21E	1.200316	0.692883	0.011566	0.073*	0.653 (15)
C22D	1.1633 (10)	0.6110 (5)	−0.0105 (6)	0.085 (4)	0.653 (15)
H22E	1.246292	0.584690	−0.001506	0.102*	0.653 (15)
C23D	1.0684 (8)	0.5845 (4)	−0.0306 (5)	0.074 (3)	0.653 (15)
H23E	1.087546	0.539982	−0.035108	0.088*	0.653 (15)
C24D	0.9478 (9)	0.6212 (6)	−0.0441 (5)	0.071 (3)	0.653 (15)
H24E	0.883543	0.602240	−0.057484	0.085*	0.653 (15)
C25D	0.9208 (10)	0.6879 (6)	−0.0376 (7)	0.054 (3)	0.653 (15)
H25E	0.836706	0.713090	−0.045780	0.065*	0.653 (15)
S1Y	0.8920 (8)	0.7789 (5)	0.0574 (5)	0.0813 (19)	0.347 (15)
C19Y	1.0241 (12)	0.7814 (8)	0.0076 (8)	0.054 (4)	0.347 (15)
H19G	1.013928	0.826836	−0.022623	0.065*	0.347 (15)
H19H	1.108093	0.776286	0.025658	0.065*	0.347 (15)
C20Y	1.0216 (16)	0.7214 (10)	−0.0157 (9)	0.044 (8)	0.347 (15)
C21Y	1.1406 (16)	0.6813 (10)	−0.0196 (13)	0.056 (6)	0.347 (15)
H21D	1.217987	0.693653	−0.009528	0.067*	0.347 (15)
C22Y	1.1474 (18)	0.6241 (9)	−0.0378 (9)	0.077 (6)	0.347 (15)
H22D	1.226880	0.594744	−0.036620	0.092*	0.347 (15)
C23Y	1.036 (2)	0.6105 (11)	−0.0579 (9)	0.090 (7)	0.347 (15)
H23D	1.038945	0.571969	−0.070840	0.109*	0.347 (15)
C24Y	0.9215 (19)	0.6529 (12)	−0.0588 (9)	0.068 (5)	0.347 (15)
H24D	0.845672	0.642475	−0.071739	0.082*	0.347 (15)
C25Y	0.913 (2)	0.7121 (10)	−0.0411 (12)	0.051 (4)	0.347 (15)
H25D	0.836440	0.744372	−0.046356	0.061*	0.347 (15)

### Atomic displacement parameters (Å^2^)

**Table:**

	*U*^11^	*U*^22^	*U*^33^	*U*^12^	*U*^13^	*U*^23^
S1A	0.0301 (3)	0.0200 (2)	0.0393 (3)	−0.00488 (19)	0.0081 (2)	−0.0098 (2)
S2A	0.0309 (3)	0.0217 (2)	0.0313 (3)	−0.00635 (19)	0.0073 (2)	−0.0069 (2)
N1A	0.0334 (9)	0.0227 (8)	0.0307 (10)	−0.0076 (7)	0.0069 (8)	−0.0084 (7)
N2A	0.0315 (9)	0.0186 (9)	0.0308 (10)	−0.0053 (7)	0.0080 (8)	−0.0071 (7)
O1A	0.0435 (10)	0.0347 (9)	0.0599 (12)	0.0027 (7)	0.0104 (9)	−0.0222 (8)
O2A	0.0482 (10)	0.0340 (9)	0.0518 (12)	−0.0091 (8)	−0.0051 (9)	0.0102 (8)
C1A	0.0329 (11)	0.0358 (12)	0.0340 (12)	−0.0105 (9)	0.0066 (9)	−0.0164 (10)
C2A	0.0289 (11)	0.0418 (13)	0.0427 (14)	−0.0057 (9)	0.0079 (10)	−0.0220 (11)
C3A	0.0355 (12)	0.0335 (12)	0.0360 (13)	0.0008 (9)	0.0002 (10)	−0.0160 (10)
C4A	0.0425 (13)	0.0291 (11)	0.0336 (13)	−0.0061 (9)	0.0085 (10)	−0.0096 (10)
C5A	0.0345 (11)	0.0310 (11)	0.0292 (12)	−0.0043 (9)	0.0079 (9)	−0.0114 (9)
C6A	0.0308 (11)	0.0302 (11)	0.0236 (11)	−0.0048 (8)	0.0012 (9)	−0.0115 (9)
C7A	0.0345 (11)	0.0278 (11)	0.0247 (11)	−0.0080 (9)	0.0037 (9)	−0.0105 (9)
C8A	0.0357 (11)	0.0243 (10)	0.0255 (11)	−0.0065 (8)	0.0054 (9)	−0.0066 (9)
C9A	0.0334 (11)	0.0264 (10)	0.0248 (11)	−0.0044 (8)	0.0031 (9)	−0.0091 (9)
C10A	0.0355 (11)	0.0252 (10)	0.0288 (11)	−0.0063 (9)	0.0078 (9)	−0.0088 (9)
C11A	0.0356 (12)	0.0279 (11)	0.0344 (12)	−0.0074 (9)	0.0069 (10)	−0.0100 (9)
C12A	0.0370 (12)	0.0236 (10)	0.0344 (12)	−0.0033 (9)	0.0035 (10)	−0.0089 (9)
C13A	0.0355 (12)	0.0296 (11)	0.0458 (15)	−0.0045 (9)	−0.0003 (11)	−0.0102 (10)
C14A	0.0423 (13)	0.0287 (12)	0.0438 (15)	0.0006 (10)	−0.0083 (11)	−0.0043 (10)
C15A	0.0448 (13)	0.0235 (11)	0.0390 (14)	−0.0051 (9)	0.0014 (11)	−0.0015 (10)
C16A	0.0364 (12)	0.0304 (12)	0.0414 (14)	−0.0062 (9)	−0.0001 (10)	−0.0056 (10)
C17A	0.0369 (12)	0.0258 (11)	0.0335 (12)	−0.0035 (9)	−0.0018 (10)	−0.0036 (9)
C18A	0.0306 (11)	0.0233 (10)	0.0258 (11)	−0.0038 (8)	0.0009 (9)	−0.0090 (8)
C19A	0.0344 (12)	0.0324 (12)	0.0737 (19)	−0.0117 (10)	0.0225 (12)	−0.0260 (13)
C20A	0.0254 (10)	0.0238 (10)	0.0436 (13)	−0.0058 (8)	0.0105 (9)	−0.0119 (9)
C21A	0.0333 (12)	0.0342 (12)	0.0445 (15)	0.0024 (9)	−0.0027 (11)	−0.0050 (11)
C22A	0.0421 (13)	0.0490 (15)	0.0387 (14)	−0.0095 (11)	−0.0008 (11)	−0.0183 (12)
C23A	0.0412 (13)	0.0300 (12)	0.0452 (14)	−0.0088 (10)	0.0116 (11)	−0.0193 (11)
C24A	0.0448 (13)	0.0256 (11)	0.0402 (14)	0.0024 (10)	0.0022 (11)	−0.0064 (10)
C25A	0.0358 (12)	0.0361 (12)	0.0320 (12)	−0.0030 (9)	0.0021 (10)	−0.0138 (10)
C26A	0.0396 (15)	0.0581 (18)	0.103 (3)	−0.0019 (13)	0.0169 (16)	−0.0499 (19)
C27A	0.0535 (17)	0.0412 (15)	0.0609 (19)	−0.0163 (12)	−0.0006 (14)	0.0159 (13)
S1B	0.0543 (3)	0.0217 (3)	0.0302 (3)	−0.0108 (2)	0.0017 (3)	−0.0054 (2)
S2B	0.0332 (3)	0.0219 (2)	0.0328 (3)	−0.0084 (2)	−0.0018 (2)	−0.0040 (2)
N2B	0.0628 (14)	0.0239 (10)	0.0314 (11)	−0.0189 (9)	0.0156 (10)	−0.0112 (8)
O1B	0.0473 (11)	0.0752 (14)	0.0357 (10)	0.0109 (10)	−0.0045 (8)	−0.0181 (9)
O2B	0.0474 (10)	0.0287 (8)	0.0323 (9)	−0.0060 (7)	−0.0001 (7)	−0.0015 (7)
C18B	0.0459 (13)	0.0242 (10)	0.0245 (11)	−0.0113 (9)	−0.0045 (10)	−0.0072 (9)
C19B	0.0366 (13)	0.0252 (12)	0.0653 (18)	−0.0047 (9)	0.0082 (12)	−0.0087 (11)
C20B	0.0368 (12)	0.0258 (11)	0.0392 (13)	−0.0058 (9)	0.0057 (10)	−0.0032 (10)
C21B	0.0485 (15)	0.0496 (15)	0.0412 (15)	−0.0040 (12)	0.0010 (12)	−0.0063 (12)
C22B	0.0634 (19)	0.073 (2)	0.0441 (17)	−0.0195 (16)	0.0089 (14)	−0.0273 (15)
C23B	0.075 (2)	0.0416 (15)	0.0479 (17)	−0.0237 (14)	0.0275 (15)	−0.0219 (13)
C24B	0.0726 (19)	0.0271 (12)	0.0473 (16)	0.0031 (12)	0.0079 (14)	−0.0067 (11)
C25B	0.0520 (15)	0.0334 (13)	0.0436 (15)	0.0029 (11)	−0.0059 (12)	−0.0111 (11)
C26B	0.0617 (18)	0.070 (2)	0.0434 (17)	0.0083 (15)	−0.0038 (14)	−0.0234 (15)
C27B	0.0524 (15)	0.0433 (14)	0.0417 (15)	−0.0127 (12)	0.0022 (12)	−0.0095 (12)
S1C	0.0381 (3)	0.0262 (3)	0.0332 (3)	−0.0085 (2)	0.0095 (2)	−0.0076 (2)
S2C	0.0535 (4)	0.0247 (3)	0.0479 (4)	−0.0063 (2)	0.0203 (3)	−0.0055 (3)
N1C	0.0368 (10)	0.0285 (9)	0.0274 (10)	−0.0124 (8)	0.0036 (8)	−0.0062 (8)
N2C	0.0369 (10)	0.0225 (9)	0.0307 (10)	−0.0068 (7)	0.0037 (8)	−0.0051 (8)
O1C	0.0427 (9)	0.0333 (9)	0.0479 (11)	−0.0034 (7)	0.0099 (8)	−0.0113 (8)
O2C	0.0723 (14)	0.0424 (11)	0.0485 (12)	−0.0232 (10)	0.0037 (10)	0.0108 (9)
C1C	0.0398 (13)	0.0328 (12)	0.0324 (13)	−0.0032 (10)	0.0070 (10)	0.0007 (10)
C2C	0.0378 (12)	0.0419 (13)	0.0269 (12)	−0.0038 (10)	0.0039 (10)	−0.0047 (10)
C3C	0.0304 (11)	0.0306 (11)	0.0341 (12)	−0.0024 (9)	−0.0026 (9)	−0.0081 (9)
C4C	0.0413 (13)	0.0323 (12)	0.0354 (13)	−0.0034 (10)	0.0087 (11)	0.0022 (10)
C5C	0.0380 (12)	0.0365 (12)	0.0293 (12)	−0.0009 (10)	0.0068 (10)	−0.0014 (10)
C6C	0.0299 (11)	0.0331 (11)	0.0290 (12)	−0.0068 (9)	0.0004 (9)	−0.0056 (9)
C7C	0.0364 (12)	0.0270 (11)	0.0339 (12)	−0.0079 (9)	0.0025 (10)	−0.0032 (9)
C8C	0.0389 (12)	0.0277 (11)	0.0235 (11)	−0.0112 (9)	0.0031 (9)	−0.0039 (9)
C9C	0.0327 (11)	0.0291 (11)	0.0280 (11)	−0.0077 (9)	0.0017 (9)	−0.0092 (9)
C10C	0.0339 (11)	0.0321 (12)	0.0265 (11)	−0.0062 (9)	0.0078 (9)	−0.0063 (9)
C11C	0.0356 (12)	0.0317 (12)	0.0271 (12)	−0.0077 (9)	0.0042 (9)	−0.0071 (9)
C12C	0.0311 (11)	0.0310 (11)	0.0316 (12)	−0.0081 (9)	0.0002 (9)	−0.0052 (9)
C13C	0.0532 (15)	0.0320 (12)	0.0350 (13)	−0.0111 (11)	−0.0006 (11)	−0.0093 (10)
C14C	0.0563 (16)	0.0306 (12)	0.0463 (16)	−0.0123 (11)	−0.0056 (12)	−0.0066 (11)
C15C	0.0431 (14)	0.0374 (13)	0.0394 (14)	−0.0152 (11)	−0.0012 (11)	0.0034 (11)
C16C	0.0387 (13)	0.0445 (14)	0.0328 (13)	−0.0083 (10)	0.0061 (10)	−0.0030 (11)
C17C	0.0333 (11)	0.0330 (12)	0.0331 (13)	−0.0055 (9)	0.0036 (10)	−0.0065 (10)
C18C	0.0307 (11)	0.0403 (13)	0.0373 (13)	−0.0051 (9)	−0.0026 (10)	−0.0196 (10)
C19C	0.0370 (12)	0.0304 (11)	0.0377 (13)	−0.0084 (9)	0.0116 (10)	−0.0090 (10)
C20C	0.0329 (11)	0.0272 (11)	0.0285 (11)	−0.0073 (9)	0.0076 (9)	−0.0057 (9)
C21C	0.0338 (12)	0.0352 (13)	0.0499 (15)	−0.0066 (10)	−0.0005 (11)	−0.0106 (11)
C22C	0.0405 (14)	0.0351 (14)	0.072 (2)	0.0016 (11)	0.0072 (13)	−0.0111 (13)
C23C	0.0533 (16)	0.0315 (13)	0.0578 (17)	−0.0107 (11)	0.0216 (13)	−0.0182 (12)
C24C	0.0499 (15)	0.0432 (14)	0.0460 (15)	−0.0161 (11)	0.0081 (12)	−0.0226 (12)
C25C	0.0337 (12)	0.0391 (13)	0.0386 (14)	−0.0047 (10)	0.0020 (10)	−0.0106 (11)
C26C	0.0379 (13)	0.0456 (14)	0.0449 (15)	−0.0023 (11)	0.0025 (11)	−0.0192 (12)
C27C	0.105 (3)	0.0408 (17)	0.071 (2)	−0.0374 (18)	−0.011 (2)	0.0111 (15)
S2D	0.0492 (4)	0.0477 (4)	0.0563 (4)	−0.0066 (3)	0.0186 (3)	−0.0207 (3)
N1D	0.0320 (10)	0.0413 (11)	0.0354 (11)	−0.0110 (8)	−0.0014 (8)	−0.0079 (9)
N2D	0.0332 (10)	0.0341 (11)	0.0376 (11)	−0.0052 (8)	0.0027 (8)	−0.0106 (9)
O1D	0.0450 (10)	0.0380 (9)	0.0616 (12)	−0.0128 (8)	0.0166 (9)	−0.0233 (9)
O2D	0.0618 (12)	0.0322 (9)	0.0441 (11)	−0.0108 (8)	0.0040 (9)	−0.0033 (8)
C1D	0.0349 (12)	0.0310 (12)	0.0423 (14)	−0.0131 (9)	0.0062 (10)	−0.0098 (10)
C2D	0.0347 (12)	0.0392 (13)	0.0432 (14)	−0.0128 (10)	0.0095 (11)	−0.0124 (11)
C3D	0.0416 (13)	0.0306 (11)	0.0343 (13)	−0.0102 (10)	0.0081 (10)	−0.0109 (10)
C4D	0.0401 (13)	0.0372 (13)	0.0425 (14)	−0.0190 (10)	0.0108 (11)	−0.0129 (11)
C5D	0.0349 (12)	0.0365 (12)	0.0361 (13)	−0.0130 (10)	0.0101 (10)	−0.0098 (10)
C6D	0.0341 (11)	0.0335 (11)	0.0232 (11)	−0.0119 (9)	0.0021 (9)	−0.0045 (9)
C7D	0.0321 (11)	0.0299 (11)	0.0253 (11)	−0.0101 (9)	0.0016 (9)	−0.0044 (9)
C8D	0.0339 (11)	0.0347 (12)	0.0224 (11)	−0.0112 (9)	0.0008 (9)	−0.0039 (9)
C9D	0.0268 (11)	0.0372 (12)	0.0291 (12)	−0.0045 (9)	−0.0015 (9)	−0.0107 (10)
C10D	0.0300 (11)	0.0374 (12)	0.0298 (12)	−0.0061 (9)	−0.0007 (9)	−0.0069 (10)
C11D	0.0400 (13)	0.0374 (12)	0.0301 (12)	−0.0033 (10)	−0.0059 (10)	−0.0109 (10)
C12D	0.0401 (13)	0.0345 (12)	0.0321 (13)	−0.0044 (10)	−0.0061 (10)	−0.0071 (10)
C13D	0.0727 (19)	0.0376 (13)	0.0355 (14)	−0.0083 (12)	−0.0067 (13)	−0.0132 (11)
C14D	0.0716 (19)	0.0338 (13)	0.0421 (15)	−0.0105 (12)	−0.0076 (13)	−0.0111 (11)
C15D	0.0424 (13)	0.0351 (13)	0.0419 (14)	−0.0062 (10)	−0.0050 (11)	−0.0072 (11)
C16D	0.0382 (13)	0.0396 (13)	0.0360 (13)	−0.0058 (10)	0.0048 (10)	−0.0101 (11)
C17D	0.0334 (12)	0.0319 (12)	0.0401 (13)	−0.0034 (9)	0.0005 (10)	−0.0111 (10)
C18D	0.0307 (12)	0.0566 (16)	0.0632 (18)	0.0055 (11)	−0.0055 (12)	−0.0428 (14)
C26D	0.0476 (15)	0.0446 (15)	0.073 (2)	−0.0082 (12)	0.0203 (14)	−0.0268 (14)
C27D	0.073 (2)	0.0348 (14)	0.0557 (18)	−0.0164 (13)	−0.0096 (15)	−0.0062 (12)
C1B	0.034 (4)	0.028 (5)	0.017 (3)	−0.006 (3)	−0.003 (3)	0.003 (3)
C2B	0.031 (3)	0.036 (6)	0.028 (4)	−0.006 (5)	0.003 (3)	−0.017 (4)
C3B	0.031 (3)	0.038 (5)	0.026 (3)	−0.013 (4)	0.0024 (18)	−0.013 (3)
C4B	0.029 (2)	0.029 (4)	0.028 (3)	−0.005 (4)	0.0014 (18)	−0.007 (3)
C5B	0.025 (3)	0.032 (5)	0.018 (3)	−0.004 (4)	0.001 (2)	−0.005 (3)
C6B	0.023 (3)	0.023 (4)	0.014 (4)	−0.005 (2)	−0.002 (3)	−0.001 (3)
C7B	0.026 (3)	0.028 (4)	0.027 (3)	−0.010 (2)	0.006 (2)	−0.008 (2)
C8B	0.032 (3)	0.024 (3)	0.026 (3)	−0.007 (2)	−0.0022 (19)	−0.006 (2)
N1B	0.034 (2)	0.0267 (18)	0.028 (2)	−0.0109 (19)	0.0058 (19)	−0.0091 (15)
C9B	0.028 (2)	0.028 (2)	0.029 (2)	−0.009 (2)	0.0063 (18)	−0.0129 (17)
C10B	0.034 (2)	0.0270 (18)	0.029 (2)	−0.0110 (15)	0.0112 (16)	−0.0103 (16)
C11B	0.032 (2)	0.026 (2)	0.037 (2)	−0.0098 (19)	0.0076 (16)	−0.0130 (19)
C12B	0.031 (3)	0.0264 (19)	0.031 (2)	−0.0069 (18)	0.0028 (19)	−0.0100 (17)
C13B	0.029 (3)	0.032 (2)	0.040 (2)	−0.004 (2)	0.000 (2)	−0.0135 (18)
C14B	0.032 (4)	0.026 (3)	0.034 (3)	−0.004 (3)	−0.009 (3)	−0.007 (2)
C15B	0.036 (5)	0.020 (3)	0.026 (3)	−0.007 (3)	−0.005 (3)	−0.005 (2)
C16B	0.025 (3)	0.035 (3)	0.035 (3)	−0.011 (2)	0.003 (3)	−0.012 (2)
C17B	0.031 (3)	0.027 (2)	0.030 (2)	−0.005 (2)	−0.003 (2)	−0.0031 (16)
C1X	0.028 (7)	0.021 (8)	0.009 (5)	0.000 (5)	−0.003 (4)	0.000 (5)
C2X	0.047 (8)	0.016 (9)	0.022 (6)	−0.011 (7)	0.003 (4)	−0.001 (4)
C3X	0.019 (4)	0.019 (7)	0.053 (8)	−0.005 (5)	0.004 (4)	−0.017 (5)
C4X	0.031 (6)	0.027 (10)	0.021 (5)	−0.011 (9)	−0.001 (4)	−0.005 (6)
C5X	0.027 (6)	0.019 (8)	0.028 (9)	−0.001 (6)	−0.004 (5)	−0.003 (7)
C6X	0.027 (7)	0.018 (8)	0.014 (6)	−0.006 (5)	−0.010 (4)	0.011 (5)
C7X	0.030 (7)	0.025 (6)	0.044 (7)	−0.007 (4)	−0.001 (5)	−0.020 (5)
C8X	0.025 (6)	0.022 (7)	0.016 (5)	−0.010 (4)	0.004 (4)	0.001 (4)
N1X	0.032 (5)	0.023 (3)	0.030 (4)	−0.009 (4)	0.008 (4)	−0.012 (3)
C9X	0.027 (5)	0.025 (4)	0.033 (5)	−0.010 (4)	0.006 (4)	−0.017 (3)
C10X	0.032 (5)	0.027 (5)	0.035 (5)	−0.002 (3)	0.000 (3)	−0.014 (4)
C11X	0.035 (5)	0.027 (4)	0.035 (5)	0.003 (3)	0.001 (3)	−0.013 (3)
C12X	0.031 (5)	0.026 (4)	0.026 (4)	−0.008 (4)	0.010 (4)	−0.010 (3)
C13X	0.034 (6)	0.025 (5)	0.037 (5)	−0.011 (4)	0.007 (5)	−0.011 (4)
C14X	0.043 (11)	0.041 (7)	0.028 (5)	0.010 (7)	−0.018 (7)	−0.006 (4)
C15X	0.047 (12)	0.034 (8)	0.044 (9)	−0.015 (7)	0.007 (8)	−0.020 (7)
C16X	0.028 (6)	0.015 (4)	0.034 (5)	−0.006 (4)	0.008 (5)	0.000 (3)
C17X	0.034 (6)	0.028 (4)	0.036 (5)	0.000 (4)	0.002 (4)	−0.008 (3)
S1D	0.0330 (8)	0.0158 (8)	0.0538 (12)	0.0001 (6)	0.0177 (7)	−0.0066 (7)
C19D	0.046 (4)	0.040 (3)	0.061 (4)	−0.007 (3)	0.028 (3)	−0.012 (3)
C20D	0.041 (7)	0.025 (4)	0.055 (6)	0.000 (4)	0.004 (4)	−0.001 (4)
C21D	0.043 (4)	0.054 (5)	0.088 (10)	0.013 (4)	−0.018 (4)	−0.034 (6)
C22D	0.057 (4)	0.044 (4)	0.149 (11)	0.006 (3)	−0.009 (5)	−0.029 (5)
C23D	0.070 (4)	0.052 (4)	0.110 (7)	−0.029 (3)	0.043 (5)	−0.039 (4)
C24D	0.071 (6)	0.087 (7)	0.069 (6)	−0.054 (5)	0.016 (4)	−0.030 (5)
C25D	0.036 (3)	0.046 (6)	0.066 (5)	−0.007 (4)	−0.001 (3)	0.001 (6)
S1Y	0.040 (2)	0.055 (3)	0.114 (4)	−0.0129 (18)	0.018 (2)	0.020 (3)
C19Y	0.028 (5)	0.041 (6)	0.098 (12)	−0.013 (4)	0.019 (6)	−0.030 (8)
C20Y	0.029 (11)	0.061 (13)	0.053 (11)	−0.025 (8)	0.028 (8)	−0.032 (9)
C21Y	0.050 (8)	0.041 (7)	0.075 (14)	−0.031 (7)	−0.004 (6)	−0.006 (6)
C22Y	0.070 (9)	0.039 (7)	0.120 (16)	0.013 (6)	−0.013 (9)	−0.030 (9)
C23Y	0.133 (19)	0.078 (12)	0.088 (13)	−0.067 (13)	0.035 (12)	−0.048 (10)
C24Y	0.082 (10)	0.080 (13)	0.059 (10)	−0.054 (10)	0.020 (7)	−0.028 (10)
C25Y	0.043 (7)	0.041 (10)	0.055 (7)	−0.007 (6)	−0.007 (5)	0.003 (9)

### Geometric parameters (Å, º)

**Table:**

S1A—C18A	1.760 (2)	C24C—H24C	0.9300
S1A—C19A	1.814 (2)	C25C—H25C	0.9300
S2A—C18A	1.661 (2)	C26C—H26G	0.9600
N1A—C9A	1.298 (3)	C26C—H26H	0.9600
N1A—N2A	1.371 (3)	C26C—H26I	0.9600
N2A—C18A	1.344 (3)	C27C—H27G	0.9600
N2A—H1N2	0.81 (3)	C27C—H27H	0.9600
O1A—C3A	1.367 (3)	C27C—H27I	0.9600
O1A—C26A	1.414 (3)	S2D—C18D	1.621 (3)
O2A—C15A	1.369 (3)	N1D—C9D	1.317 (3)
O2A—C27A	1.435 (3)	N1D—N2D	1.354 (3)
C1A—C6A	1.384 (3)	N2D—C18D	1.367 (3)
C1A—C2A	1.391 (3)	N2D—H4N2	0.91 (4)
C1A—H1AA	0.9300	O1D—C3D	1.367 (3)
C2A—C3A	1.386 (3)	O1D—C26D	1.428 (3)
C2A—H2AA	0.9300	O2D—C15D	1.366 (3)
C3A—C4A	1.389 (3)	O2D—C27D	1.434 (3)
C4A—C5A	1.375 (3)	C1D—C2D	1.382 (3)
C4A—H4AA	0.9300	C1D—C6D	1.393 (3)
C5A—C6A	1.405 (3)	C1D—H1DA	0.9300
C5A—H5AA	0.9300	C2D—C3D	1.391 (3)
C6A—C7A	1.464 (3)	C2D—H2DA	0.9300
C7A—C8A	1.332 (3)	C3D—C4D	1.391 (3)
C7A—H7AA	0.9300	C4D—C5D	1.373 (3)
C8A—C9A	1.462 (3)	C4D—H4DA	0.9300
C8A—H8AA	0.9300	C5D—C6D	1.402 (3)
C9A—C10A	1.476 (3)	C5D—H5DA	0.9300
C10A—C11A	1.337 (3)	C6D—C7D	1.458 (3)
C10A—H10A	0.9300	C7D—C8D	1.340 (3)
C11A—C12A	1.467 (3)	C7D—H7DA	0.9300
C11A—H11A	0.9300	C8D—C9D	1.463 (3)
C12A—C17A	1.397 (3)	C8D—H8DA	0.9300
C12A—C13A	1.398 (3)	C9D—C10D	1.471 (3)
C13A—C14A	1.380 (3)	C10D—C11D	1.337 (3)
C13A—H13A	0.9300	C10D—H10D	0.9300
C14A—C15A	1.392 (4)	C11D—C12D	1.461 (3)
C14A—H14A	0.9300	C11D—H11D	0.9300
C15A—C16A	1.390 (3)	C12D—C13D	1.395 (3)
C16A—C17A	1.390 (3)	C12D—C17D	1.403 (4)
C16A—H16A	0.9300	C13D—C14D	1.380 (4)
C17A—H17A	0.9300	C13D—H13D	0.9300
C19A—C20A	1.508 (3)	C14D—C15D	1.385 (4)
C19A—H19A	0.9700	C14D—H14D	0.9300
C19A—H19B	0.9700	C15D—C16D	1.396 (4)
C20A—C21A	1.383 (4)	C16D—C17D	1.378 (3)
C20A—C25A	1.391 (3)	C16D—H16D	0.9300
C21A—C22A	1.381 (4)	C17D—H17D	0.9300
C21A—H21A	0.9300	C18D—S1D	1.709 (4)
C22A—C23A	1.378 (4)	C18D—S1Y	2.032 (11)
C22A—H22A	0.9300	C26D—H26J	0.9600
C23A—C24A	1.372 (4)	C26D—H26K	0.9600
C23A—H23A	0.9300	C26D—H26L	0.9600
C24A—C25A	1.389 (3)	C27D—H27J	0.9600
C24A—H24A	0.9300	C27D—H27K	0.9600
C25A—H25A	0.9300	C27D—H27L	0.9600
C26A—H26A	0.9600	C1B—C2B	1.382 (9)
C26A—H26B	0.9600	C1B—C6B	1.396 (7)
C26A—H26C	0.9600	C1B—H1BA	0.9300
C27A—H27A	0.9600	C2B—C3B	1.384 (8)
C27A—H27B	0.9600	C2B—H2BA	0.9300
C27A—H27C	0.9600	C3B—C4B	1.397 (8)
S1B—C18B	1.755 (2)	C4B—C5B	1.381 (7)
S1B—C19B	1.804 (3)	C4B—H4BA	0.9300
S2B—C18B	1.657 (2)	C5B—C6B	1.396 (7)
N2B—C18B	1.348 (3)	C5B—H5BA	0.9300
N2B—N1X	1.393 (9)	C6B—C7B	1.454 (7)
N2B—N1B	1.402 (5)	C7B—C8B	1.342 (7)
N2B—H2N2	0.82 (3)	C7B—H7BA	0.9300
O1B—C3B	1.248 (9)	C8B—C9B	1.466 (6)
O1B—C26B	1.423 (3)	C8B—H8BA	0.9300
O1B—C3X	1.613 (16)	N1B—C9B	1.292 (6)
O2B—C15X	1.309 (19)	C9B—C10B	1.473 (5)
O2B—C15B	1.406 (9)	C10B—C11B	1.325 (6)
O2B—C27B	1.426 (3)	C10B—H10B	0.9300
C19B—C20B	1.509 (3)	C11B—C12B	1.468 (6)
C19B—H19C	0.9700	C11B—H11B	0.9300
C19B—H19D	0.9700	C12B—C13B	1.394 (7)
C20B—C21B	1.375 (4)	C12B—C17B	1.396 (6)
C20B—C25B	1.384 (3)	C13B—C14B	1.390 (7)
C21B—C22B	1.381 (4)	C13B—H13B	0.9300
C21B—H21B	0.9300	C14B—C15B	1.395 (9)
C22B—C23B	1.369 (5)	C14B—H14B	0.9300
C22B—H22B	0.9300	C15B—C16B	1.384 (8)
C23B—C24B	1.372 (4)	C16B—C17B	1.389 (7)
C23B—H23B	0.9300	C16B—H16B	0.9300
C24B—C25B	1.388 (4)	C17B—H17B	0.9300
C24B—H24B	0.9300	C1X—C2X	1.390 (14)
C25B—H25B	0.9300	C1X—C6X	1.400 (13)
C26B—H26D	0.9600	C1X—H1XB	0.9300
C26B—H26E	0.9600	C2X—C3X	1.385 (14)
C26B—H26F	0.9600	C2X—H2XB	0.9300
C27B—H27D	0.9600	C3X—C4X	1.403 (14)
C27B—H27E	0.9600	C4X—C5X	1.377 (12)
C27B—H27F	0.9600	C4X—H4XB	0.9300
S1C—C18C	1.763 (2)	C5X—C6X	1.412 (12)
S1C—C19C	1.825 (2)	C5X—H5XB	0.9300
S2C—C18C	1.648 (3)	C6X—C7X	1.456 (12)
N1C—C9C	1.314 (3)	C7X—C8X	1.336 (12)
N1C—N2C	1.362 (3)	C7X—H7XB	0.9300
N2C—C18C	1.352 (3)	C8X—C9X	1.463 (11)
N2C—H3N2	0.91 (3)	C8X—H8XB	0.9300
O1C—C3C	1.370 (3)	N1X—C9X	1.284 (11)
O1C—C26C	1.427 (3)	C9X—C10X	1.482 (11)
O2C—C15C	1.366 (3)	C10X—C11X	1.321 (10)
O2C—C27C	1.436 (4)	C10X—H10E	0.9300
C1C—C2C	1.380 (3)	C11X—C12X	1.486 (9)
C1C—C6C	1.389 (3)	C11X—H11E	0.9300
C1C—H1CA	0.9300	C12X—C17X	1.379 (11)
C2C—C3C	1.382 (3)	C12X—C13X	1.387 (12)
C2C—H2CA	0.9300	C13X—C14X	1.390 (13)
C3C—C4C	1.386 (3)	C13X—H13E	0.9300
C4C—C5C	1.375 (3)	C14X—C15X	1.387 (15)
C4C—H4CA	0.9300	C14X—H14E	0.9300
C5C—C6C	1.400 (3)	C15X—C16X	1.378 (14)
C5C—H5CA	0.9300	C16X—C17X	1.388 (13)
C6C—C7C	1.466 (3)	C16X—H16E	0.9300
C7C—C8C	1.331 (3)	C17X—H17E	0.9300
C7C—H7CA	0.9300	S1D—C19D	1.841 (6)
C8C—C9C	1.460 (3)	C19D—C20D	1.520 (8)
C8C—H8CA	0.9300	C19D—H19I	0.9700
C9C—C10C	1.476 (3)	C19D—H19J	0.9700
C10C—C11C	1.324 (3)	C20D—C25D	1.375 (10)
C10C—H10C	0.9300	C20D—C21D	1.388 (8)
C11C—C12C	1.471 (3)	C21D—C22D	1.358 (9)
C11C—H11C	0.9300	C21D—H21E	0.9300
C12C—C13C	1.392 (3)	C22D—C23D	1.359 (11)
C12C—C17C	1.398 (3)	C22D—H22E	0.9300
C13C—C14C	1.393 (4)	C23D—C24D	1.342 (13)
C13C—H13C	0.9300	C23D—H23E	0.9300
C14C—C15C	1.373 (4)	C24D—C25D	1.390 (11)
C14C—H14C	0.9300	C24D—H24E	0.9300
C15C—C16C	1.394 (4)	C25D—H25E	0.9300
C16C—C17C	1.376 (3)	S1Y—C19Y	1.785 (12)
C16C—H16C	0.9300	C19Y—C20Y	1.508 (13)
C17C—H17C	0.9300	C19Y—H19G	0.9700
C19C—C20C	1.506 (3)	C19Y—H19H	0.9700
C19C—H19E	0.9700	C20Y—C25Y	1.373 (14)
C19C—H19F	0.9700	C20Y—C21Y	1.378 (13)
C20C—C25C	1.390 (3)	C21Y—C22Y	1.362 (13)
C20C—C21C	1.391 (3)	C21Y—H21D	0.9300
C21C—C22C	1.384 (4)	C22Y—C23Y	1.367 (16)
C21C—H21C	0.9300	C22Y—H22D	0.9300
C22C—C23C	1.375 (4)	C23Y—C24Y	1.341 (18)
C22C—H22C	0.9300	C23Y—H23D	0.9300
C23C—C24C	1.369 (4)	C24Y—C25Y	1.389 (15)
C23C—H23C	0.9300	C24Y—H24D	0.9300
C24C—C25C	1.385 (3)	C25Y—H25D	0.9300
			
C18A—S1A—C19A	101.27 (10)	H26H—C26C—H26I	109.5
C9A—N1A—N2A	120.07 (17)	O2C—C27C—H27G	109.5
C18A—N2A—N1A	118.15 (17)	O2C—C27C—H27H	109.5
C18A—N2A—H1N2	115.8 (19)	H27G—C27C—H27H	109.5
N1A—N2A—H1N2	126.1 (19)	O2C—C27C—H27I	109.5
C3A—O1A—C26A	118.0 (2)	H27G—C27C—H27I	109.5
C15A—O2A—C27A	117.6 (2)	H27H—C27C—H27I	109.5
C6A—C1A—C2A	122.6 (2)	C9D—N1D—N2D	117.5 (2)
C6A—C1A—H1AA	118.7	N1D—N2D—C18D	119.7 (2)
C2A—C1A—H1AA	118.7	N1D—N2D—H4N2	125 (2)
C3A—C2A—C1A	118.7 (2)	C18D—N2D—H4N2	115 (2)
C3A—C2A—H2AA	120.6	C3D—O1D—C26D	117.54 (19)
C1A—C2A—H2AA	120.6	C15D—O2D—C27D	117.7 (2)
O1A—C3A—C2A	125.1 (2)	C2D—C1D—C6D	122.9 (2)
O1A—C3A—C4A	115.0 (2)	C2D—C1D—H1DA	118.5
C2A—C3A—C4A	119.8 (2)	C6D—C1D—H1DA	118.5
C5A—C4A—C3A	120.7 (2)	C1D—C2D—C3D	118.7 (2)
C5A—C4A—H4AA	119.7	C1D—C2D—H2DA	120.7
C3A—C4A—H4AA	119.7	C3D—C2D—H2DA	120.7
C4A—C5A—C6A	120.8 (2)	O1D—C3D—C4D	116.1 (2)
C4A—C5A—H5AA	119.6	O1D—C3D—C2D	124.2 (2)
C6A—C5A—H5AA	119.6	C4D—C3D—C2D	119.7 (2)
C1A—C6A—C5A	117.3 (2)	C5D—C4D—C3D	120.6 (2)
C1A—C6A—C7A	119.8 (2)	C5D—C4D—H4DA	119.7
C5A—C6A—C7A	122.84 (19)	C3D—C4D—H4DA	119.7
C8A—C7A—C6A	127.2 (2)	C4D—C5D—C6D	121.2 (2)
C8A—C7A—H7AA	116.4	C4D—C5D—H5DA	119.4
C6A—C7A—H7AA	116.4	C6D—C5D—H5DA	119.4
C7A—C8A—C9A	125.1 (2)	C1D—C6D—C5D	116.8 (2)
C7A—C8A—H8AA	117.5	C1D—C6D—C7D	119.51 (19)
C9A—C8A—H8AA	117.5	C5D—C6D—C7D	123.6 (2)
N1A—C9A—C8A	125.80 (19)	C8D—C7D—C6D	128.0 (2)
N1A—C9A—C10A	114.22 (18)	C8D—C7D—H7DA	116.0
C8A—C9A—C10A	119.91 (19)	C6D—C7D—H7DA	116.0
C11A—C10A—C9A	122.3 (2)	C7D—C8D—C9D	123.8 (2)
C11A—C10A—H10A	118.9	C7D—C8D—H8DA	118.1
C9A—C10A—H10A	118.9	C9D—C8D—H8DA	118.1
C10A—C11A—C12A	126.6 (2)	N1D—C9D—C8D	126.3 (2)
C10A—C11A—H11A	116.7	N1D—C9D—C10D	114.7 (2)
C12A—C11A—H11A	116.7	C8D—C9D—C10D	118.9 (2)
C17A—C12A—C13A	117.3 (2)	C11D—C10D—C9D	125.1 (2)
C17A—C12A—C11A	123.2 (2)	C11D—C10D—H10D	117.5
C13A—C12A—C11A	119.5 (2)	C9D—C10D—H10D	117.5
C14A—C13A—C12A	121.7 (2)	C10D—C11D—C12D	125.8 (2)
C14A—C13A—H13A	119.2	C10D—C11D—H11D	117.1
C12A—C13A—H13A	119.2	C12D—C11D—H11D	117.1
C13A—C14A—C15A	119.9 (2)	C13D—C12D—C17D	116.7 (2)
C13A—C14A—H14A	120.0	C13D—C12D—C11D	120.4 (2)
C15A—C14A—H14A	120.0	C17D—C12D—C11D	122.9 (2)
O2A—C15A—C16A	124.4 (2)	C14D—C13D—C12D	122.4 (3)
O2A—C15A—C14A	115.8 (2)	C14D—C13D—H13D	118.8
C16A—C15A—C14A	119.8 (2)	C12D—C13D—H13D	118.8
C17A—C16A—C15A	119.5 (2)	C13D—C14D—C15D	119.8 (2)
C17A—C16A—H16A	120.3	C13D—C14D—H14D	120.1
C15A—C16A—H16A	120.3	C15D—C14D—H14D	120.1
C16A—C17A—C12A	121.8 (2)	O2D—C15D—C14D	125.0 (2)
C16A—C17A—H17A	119.1	O2D—C15D—C16D	115.7 (2)
C12A—C17A—H17A	119.1	C14D—C15D—C16D	119.3 (2)
N2A—C18A—S2A	121.55 (15)	C17D—C16D—C15D	120.1 (2)
N2A—C18A—S1A	112.85 (15)	C17D—C16D—H16D	120.0
S2A—C18A—S1A	125.60 (13)	C15D—C16D—H16D	120.0
C20A—C19A—S1A	109.27 (15)	C16D—C17D—C12D	121.7 (2)
C20A—C19A—H19A	109.8	C16D—C17D—H17D	119.1
S1A—C19A—H19A	109.8	C12D—C17D—H17D	119.1
C20A—C19A—H19B	109.8	N2D—C18D—S2D	122.8 (2)
S1A—C19A—H19B	109.8	N2D—C18D—S1D	112.9 (2)
H19A—C19A—H19B	108.3	S2D—C18D—S1D	124.23 (17)
C21A—C20A—C25A	118.5 (2)	N2D—C18D—S1Y	109.0 (3)
C21A—C20A—C19A	119.8 (2)	S2D—C18D—S1Y	128.0 (2)
C25A—C20A—C19A	121.7 (2)	O1D—C26D—H26J	109.5
C22A—C21A—C20A	121.2 (2)	O1D—C26D—H26K	109.5
C22A—C21A—H21A	119.4	H26J—C26D—H26K	109.5
C20A—C21A—H21A	119.4	O1D—C26D—H26L	109.5
C23A—C22A—C21A	119.9 (2)	H26J—C26D—H26L	109.5
C23A—C22A—H22A	120.1	H26K—C26D—H26L	109.5
C21A—C22A—H22A	120.1	O2D—C27D—H27J	109.5
C24A—C23A—C22A	119.8 (2)	O2D—C27D—H27K	109.5
C24A—C23A—H23A	120.1	H27J—C27D—H27K	109.5
C22A—C23A—H23A	120.1	O2D—C27D—H27L	109.5
C23A—C24A—C25A	120.6 (2)	H27J—C27D—H27L	109.5
C23A—C24A—H24A	119.7	H27K—C27D—H27L	109.5
C25A—C24A—H24A	119.7	C2B—C1B—C6B	123.5 (7)
C24A—C25A—C20A	120.0 (2)	C2B—C1B—H1BA	118.2
C24A—C25A—H25A	120.0	C6B—C1B—H1BA	118.2
C20A—C25A—H25A	120.0	C1B—C2B—C3B	118.2 (7)
O1A—C26A—H26A	109.5	C1B—C2B—H2BA	120.9
O1A—C26A—H26B	109.5	C3B—C2B—H2BA	120.9
H26A—C26A—H26B	109.5	O1B—C3B—C2B	125.1 (7)
O1A—C26A—H26C	109.5	O1B—C3B—C4B	114.9 (7)
H26A—C26A—H26C	109.5	C2B—C3B—C4B	119.8 (7)
H26B—C26A—H26C	109.5	C5B—C4B—C3B	120.8 (7)
O2A—C27A—H27A	109.5	C5B—C4B—H4BA	119.6
O2A—C27A—H27B	109.5	C3B—C4B—H4BA	119.6
H27A—C27A—H27B	109.5	C4B—C5B—C6B	120.7 (7)
O2A—C27A—H27C	109.5	C4B—C5B—H5BA	119.6
H27A—C27A—H27C	109.5	C6B—C5B—H5BA	119.6
H27B—C27A—H27C	109.5	C5B—C6B—C1B	116.8 (7)
C18B—S1B—C19B	101.73 (11)	C5B—C6B—C7B	125.0 (6)
C18B—N2B—N1X	107.4 (4)	C1B—C6B—C7B	118.1 (7)
C18B—N2B—N1B	122.2 (3)	C8B—C7B—C6B	124.7 (6)
C18B—N2B—H2N2	115.3 (19)	C8B—C7B—H7BA	117.7
N1X—N2B—H2N2	133 (2)	C6B—C7B—H7BA	117.7
N1B—N2B—H2N2	122.3 (19)	C7B—C8B—C9B	120.4 (5)
C3B—O1B—C26B	117.6 (4)	C7B—C8B—H8BA	119.8
C26B—O1B—C3X	116.9 (7)	C9B—C8B—H8BA	119.8
C15X—O2B—C27B	108.6 (9)	C9B—N1B—N2B	121.8 (4)
C15B—O2B—C27B	121.0 (4)	N1B—C9B—C8B	125.4 (4)
N2B—C18B—S2B	122.09 (17)	N1B—C9B—C10B	116.2 (4)
N2B—C18B—S1B	112.17 (16)	C8B—C9B—C10B	118.4 (4)
S2B—C18B—S1B	125.72 (14)	C11B—C10B—C9B	124.1 (4)
C20B—C19B—S1B	107.82 (16)	C11B—C10B—H10B	117.9
C20B—C19B—H19C	110.1	C9B—C10B—H10B	117.9
S1B—C19B—H19C	110.1	C10B—C11B—C12B	126.4 (4)
C20B—C19B—H19D	110.1	C10B—C11B—H11B	116.8
S1B—C19B—H19D	110.1	C12B—C11B—H11B	116.8
H19C—C19B—H19D	108.5	C13B—C12B—C17B	117.4 (4)
C21B—C20B—C25B	118.5 (2)	C13B—C12B—C11B	119.9 (4)
C21B—C20B—C19B	120.1 (2)	C17B—C12B—C11B	122.7 (4)
C25B—C20B—C19B	121.4 (2)	C14B—C13B—C12B	122.1 (5)
C20B—C21B—C22B	121.0 (3)	C14B—C13B—H13B	118.9
C20B—C21B—H21B	119.5	C12B—C13B—H13B	118.9
C22B—C21B—H21B	119.5	C13B—C14B—C15B	119.0 (7)
C23B—C22B—C21B	120.4 (3)	C13B—C14B—H14B	120.5
C23B—C22B—H22B	119.8	C15B—C14B—H14B	120.5
C21B—C22B—H22B	119.8	C16B—C15B—C14B	120.0 (7)
C22B—C23B—C24B	119.2 (3)	C16B—C15B—O2B	123.7 (8)
C22B—C23B—H23B	120.4	C14B—C15B—O2B	116.1 (6)
C24B—C23B—H23B	120.4	C15B—C16B—C17B	120.1 (6)
C23B—C24B—C25B	120.6 (3)	C15B—C16B—H16B	120.0
C23B—C24B—H24B	119.7	C17B—C16B—H16B	120.0
C25B—C24B—H24B	119.7	C16B—C17B—C12B	121.3 (4)
C20B—C25B—C24B	120.2 (3)	C16B—C17B—H17B	119.3
C20B—C25B—H25B	119.9	C12B—C17B—H17B	119.3
C24B—C25B—H25B	119.9	C2X—C1X—C6X	120.2 (13)
O1B—C26B—H26D	109.5	C2X—C1X—H1XB	119.9
O1B—C26B—H26E	109.5	C6X—C1X—H1XB	119.9
H26D—C26B—H26E	109.5	C3X—C2X—C1X	122.4 (15)
O1B—C26B—H26F	109.5	C3X—C2X—H2XB	118.8
H26D—C26B—H26F	109.5	C1X—C2X—H2XB	118.8
H26E—C26B—H26F	109.5	C2X—C3X—C4X	118.0 (14)
O2B—C27B—H27D	109.5	C2X—C3X—O1B	123.5 (13)
O2B—C27B—H27E	109.5	C4X—C3X—O1B	117.8 (13)
H27D—C27B—H27E	109.5	C5X—C4X—C3X	119.9 (13)
O2B—C27B—H27F	109.5	C5X—C4X—H4XB	120.1
H27D—C27B—H27F	109.5	C3X—C4X—H4XB	120.1
H27E—C27B—H27F	109.5	C4X—C5X—C6X	122.6 (14)
C18C—S1C—C19C	100.52 (11)	C4X—C5X—H5XB	118.7
C9C—N1C—N2C	119.70 (18)	C6X—C5X—H5XB	118.7
C18C—N2C—N1C	118.99 (19)	C1X—C6X—C5X	116.9 (12)
C18C—N2C—H3N2	110 (2)	C1X—C6X—C7X	121.1 (13)
N1C—N2C—H3N2	131 (2)	C5X—C6X—C7X	121.9 (13)
C3C—O1C—C26C	117.62 (18)	C8X—C7X—C6X	129.0 (13)
C15C—O2C—C27C	116.8 (3)	C8X—C7X—H7XB	115.5
C2C—C1C—C6C	122.4 (2)	C6X—C7X—H7XB	115.5
C2C—C1C—H1CA	118.8	C7X—C8X—C9X	123.2 (10)
C6C—C1C—H1CA	118.8	C7X—C8X—H8XB	118.4
C1C—C2C—C3C	119.4 (2)	C9X—C8X—H8XB	118.4
C1C—C2C—H2CA	120.3	C9X—N1X—N2B	111.3 (7)
C3C—C2C—H2CA	120.3	N1X—C9X—C8X	125.6 (8)
O1C—C3C—C2C	124.0 (2)	N1X—C9X—C10X	112.0 (8)
O1C—C3C—C4C	116.7 (2)	C8X—C9X—C10X	122.3 (8)
C2C—C3C—C4C	119.2 (2)	C11X—C10X—C9X	124.8 (8)
C5C—C4C—C3C	121.0 (2)	C11X—C10X—H10E	117.6
C5C—C4C—H4CA	119.5	C9X—C10X—H10E	117.6
C3C—C4C—H4CA	119.5	C10X—C11X—C12X	124.1 (8)
C4C—C5C—C6C	120.7 (2)	C10X—C11X—H11E	118.0
C4C—C5C—H5CA	119.7	C12X—C11X—H11E	118.0
C6C—C5C—H5CA	119.7	C17X—C12X—C13X	119.0 (7)
C1C—C6C—C5C	117.2 (2)	C17X—C12X—C11X	118.6 (8)
C1C—C6C—C7C	118.6 (2)	C13X—C12X—C11X	122.5 (9)
C5C—C6C—C7C	124.2 (2)	C12X—C13X—C14X	118.4 (10)
C8C—C7C—C6C	127.7 (2)	C12X—C13X—H13E	120.8
C8C—C7C—H7CA	116.2	C14X—C13X—H13E	120.8
C6C—C7C—H7CA	116.2	C15X—C14X—C13X	122.7 (13)
C7C—C8C—C9C	124.3 (2)	C15X—C14X—H14E	118.6
C7C—C8C—H8CA	117.8	C13X—C14X—H14E	118.6
C9C—C8C—H8CA	117.8	O2B—C15X—C16X	130.1 (16)
N1C—C9C—C8C	126.8 (2)	O2B—C15X—C14X	111.3 (13)
N1C—C9C—C10C	114.27 (19)	C16X—C15X—C14X	118.2 (15)
C8C—C9C—C10C	118.89 (19)	C15X—C16X—C17X	119.3 (12)
C11C—C10C—C9C	124.9 (2)	C15X—C16X—H16E	120.3
C11C—C10C—H10C	117.5	C17X—C16X—H16E	120.3
C9C—C10C—H10C	117.5	C12X—C17X—C16X	122.2 (9)
C10C—C11C—C12C	125.9 (2)	C12X—C17X—H17E	118.9
C10C—C11C—H11C	117.1	C16X—C17X—H17E	118.9
C12C—C11C—H11C	117.1	C18D—S1D—C19D	101.7 (3)
C13C—C12C—C17C	117.3 (2)	C20D—C19D—S1D	110.8 (6)
C13C—C12C—C11C	119.6 (2)	C20D—C19D—H19I	109.5
C17C—C12C—C11C	123.0 (2)	S1D—C19D—H19I	109.5
C12C—C13C—C14C	121.8 (2)	C20D—C19D—H19J	109.5
C12C—C13C—H13C	119.1	S1D—C19D—H19J	109.5
C14C—C13C—H13C	119.1	H19I—C19D—H19J	108.1
C15C—C14C—C13C	119.6 (2)	C25D—C20D—C21D	116.9 (7)
C15C—C14C—H14C	120.2	C25D—C20D—C19D	121.2 (7)
C13C—C14C—H14C	120.2	C21D—C20D—C19D	121.9 (8)
O2C—C15C—C14C	125.2 (2)	C22D—C21D—C20D	121.4 (9)
O2C—C15C—C16C	115.1 (2)	C22D—C21D—H21E	119.3
C14C—C15C—C16C	119.7 (2)	C20D—C21D—H21E	119.3
C17C—C16C—C15C	120.3 (2)	C21D—C22D—C23D	119.7 (8)
C17C—C16C—H16C	119.8	C21D—C22D—H22E	120.2
C15C—C16C—H16C	119.8	C23D—C22D—H22E	120.2
C16C—C17C—C12C	121.2 (2)	C24D—C23D—C22D	121.6 (7)
C16C—C17C—H17C	119.4	C24D—C23D—H23E	119.2
C12C—C17C—H17C	119.4	C22D—C23D—H23E	119.2
N2C—C18C—S2C	122.25 (18)	C23D—C24D—C25D	118.6 (8)
N2C—C18C—S1C	113.37 (18)	C23D—C24D—H24E	120.7
S2C—C18C—S1C	124.37 (14)	C25D—C24D—H24E	120.7
C20C—C19C—S1C	108.63 (15)	C20D—C25D—C24D	121.6 (8)
C20C—C19C—H19E	110.0	C20D—C25D—H25E	119.2
S1C—C19C—H19E	110.0	C24D—C25D—H25E	119.2
C20C—C19C—H19F	110.0	C19Y—S1Y—C18D	96.7 (5)
S1C—C19C—H19F	110.0	C20Y—C19Y—S1Y	109.0 (9)
H19E—C19C—H19F	108.3	C20Y—C19Y—H19G	109.9
C25C—C20C—C21C	118.6 (2)	S1Y—C19Y—H19G	109.9
C25C—C20C—C19C	120.9 (2)	C20Y—C19Y—H19H	109.9
C21C—C20C—C19C	120.5 (2)	S1Y—C19Y—H19H	109.9
C22C—C21C—C20C	120.5 (2)	H19G—C19Y—H19H	108.3
C22C—C21C—H21C	119.8	C25Y—C20Y—C21Y	118.4 (14)
C20C—C21C—H21C	119.8	C25Y—C20Y—C19Y	123.5 (14)
C23C—C22C—C21C	120.3 (2)	C21Y—C20Y—C19Y	117.3 (13)
C23C—C22C—H22C	119.9	C22Y—C21Y—C20Y	121.4 (13)
C21C—C22C—H22C	119.9	C22Y—C21Y—H21D	119.3
C24C—C23C—C22C	119.8 (2)	C20Y—C21Y—H21D	119.3
C24C—C23C—H23C	120.1	C21Y—C22Y—C23Y	119.0 (14)
C22C—C23C—H23C	120.1	C21Y—C22Y—H22D	120.5
C23C—C24C—C25C	120.6 (2)	C23Y—C22Y—H22D	120.5
C23C—C24C—H24C	119.7	C24Y—C23Y—C22Y	119.9 (13)
C25C—C24C—H24C	119.7	C24Y—C23Y—H23D	120.1
C24C—C25C—C20C	120.2 (2)	C22Y—C23Y—H23D	120.1
C24C—C25C—H25C	119.9	C23Y—C24Y—C25Y	121.8 (15)
C20C—C25C—H25C	119.9	C23Y—C24Y—H24D	119.1
O1C—C26C—H26G	109.5	C25Y—C24Y—H24D	119.1
O1C—C26C—H26H	109.5	C20Y—C25Y—C24Y	118.0 (15)
H26G—C26C—H26H	109.5	C20Y—C25Y—H25D	121.0
O1C—C26C—H26I	109.5	C24Y—C25Y—H25D	121.0
H26G—C26C—H26I	109.5		
			
C9A—N1A—N2A—C18A	−178.1 (2)	C2D—C1D—C6D—C5D	−0.4 (4)
C6A—C1A—C2A—C3A	−0.1 (4)	C2D—C1D—C6D—C7D	178.3 (2)
C26A—O1A—C3A—C2A	2.8 (4)	C4D—C5D—C6D—C1D	1.3 (4)
C26A—O1A—C3A—C4A	−178.7 (3)	C4D—C5D—C6D—C7D	−177.3 (2)
C1A—C2A—C3A—O1A	178.2 (2)	C1D—C6D—C7D—C8D	172.1 (2)
C1A—C2A—C3A—C4A	−0.3 (4)	C5D—C6D—C7D—C8D	−9.3 (4)
O1A—C3A—C4A—C5A	−178.0 (2)	C6D—C7D—C8D—C9D	176.0 (2)
C2A—C3A—C4A—C5A	0.6 (4)	N2D—N1D—C9D—C8D	−7.2 (3)
C3A—C4A—C5A—C6A	−0.5 (4)	N2D—N1D—C9D—C10D	177.91 (19)
C2A—C1A—C6A—C5A	0.2 (3)	C7D—C8D—C9D—N1D	156.8 (2)
C2A—C1A—C6A—C7A	−178.5 (2)	C7D—C8D—C9D—C10D	−28.4 (3)
C4A—C5A—C6A—C1A	0.1 (3)	N1D—C9D—C10D—C11D	−15.4 (3)
C4A—C5A—C6A—C7A	178.8 (2)	C8D—C9D—C10D—C11D	169.3 (2)
C1A—C6A—C7A—C8A	−175.4 (2)	C9D—C10D—C11D—C12D	172.4 (2)
C5A—C6A—C7A—C8A	5.9 (4)	C10D—C11D—C12D—C13D	167.0 (3)
C6A—C7A—C8A—C9A	−178.1 (2)	C10D—C11D—C12D—C17D	−15.9 (4)
N2A—N1A—C9A—C8A	−1.5 (3)	C17D—C12D—C13D—C14D	2.3 (4)
N2A—N1A—C9A—C10A	175.53 (19)	C11D—C12D—C13D—C14D	179.6 (3)
C7A—C8A—C9A—N1A	−175.0 (2)	C12D—C13D—C14D—C15D	−0.4 (5)
C7A—C8A—C9A—C10A	8.1 (3)	C27D—O2D—C15D—C14D	0.7 (4)
N1A—C9A—C10A—C11A	−37.5 (3)	C27D—O2D—C15D—C16D	−177.9 (2)
C8A—C9A—C10A—C11A	139.7 (2)	C13D—C14D—C15D—O2D	179.4 (3)
C9A—C10A—C11A—C12A	−176.9 (2)	C13D—C14D—C15D—C16D	−2.1 (4)
C10A—C11A—C12A—C17A	−19.6 (4)	O2D—C15D—C16D—C17D	−178.8 (2)
C10A—C11A—C12A—C13A	158.7 (2)	C14D—C15D—C16D—C17D	2.5 (4)
C17A—C12A—C13A—C14A	1.0 (4)	C15D—C16D—C17D—C12D	−0.5 (4)
C11A—C12A—C13A—C14A	−177.3 (2)	C13D—C12D—C17D—C16D	−1.9 (4)
C12A—C13A—C14A—C15A	−1.2 (4)	C11D—C12D—C17D—C16D	−179.1 (2)
C27A—O2A—C15A—C16A	0.9 (4)	N1D—N2D—C18D—S2D	178.81 (18)
C27A—O2A—C15A—C14A	−179.9 (3)	N1D—N2D—C18D—S1D	2.6 (3)
C13A—C14A—C15A—O2A	−178.6 (2)	N1D—N2D—C18D—S1Y	−6.9 (4)
C13A—C14A—C15A—C16A	0.7 (4)	C6B—C1B—C2B—C3B	3 (2)
O2A—C15A—C16A—C17A	179.0 (2)	C26B—O1B—C3B—C2B	7.3 (15)
C14A—C15A—C16A—C17A	−0.2 (4)	C26B—O1B—C3B—C4B	−177.9 (7)
C15A—C16A—C17A—C12A	0.1 (4)	C1B—C2B—C3B—O1B	173.4 (13)
C13A—C12A—C17A—C16A	−0.5 (3)	C1B—C2B—C3B—C4B	−1 (2)
C11A—C12A—C17A—C16A	177.8 (2)	O1B—C3B—C4B—C5B	−175.3 (10)
N1A—N2A—C18A—S2A	175.75 (15)	C2B—C3B—C4B—C5B	−0.2 (18)
N1A—N2A—C18A—S1A	−3.4 (3)	C3B—C4B—C5B—C6B	−0.1 (16)
C19A—S1A—C18A—N2A	−179.45 (18)	C4B—C5B—C6B—C1B	1.7 (15)
C19A—S1A—C18A—S2A	1.47 (19)	C4B—C5B—C6B—C7B	−176.6 (9)
C18A—S1A—C19A—C20A	160.53 (19)	C2B—C1B—C6B—C5B	−3 (2)
S1A—C19A—C20A—C21A	−96.9 (2)	C2B—C1B—C6B—C7B	175.2 (13)
S1A—C19A—C20A—C25A	85.0 (2)	C5B—C6B—C7B—C8B	16.4 (14)
C25A—C20A—C21A—C22A	1.1 (4)	C1B—C6B—C7B—C8B	−162.0 (9)
C19A—C20A—C21A—C22A	−177.1 (2)	C6B—C7B—C8B—C9B	178.0 (7)
C20A—C21A—C22A—C23A	−0.9 (4)	C18B—N2B—N1B—C9B	−175.6 (4)
C21A—C22A—C23A—C24A	0.1 (4)	N2B—N1B—C9B—C8B	8.6 (7)
C22A—C23A—C24A—C25A	0.5 (4)	N2B—N1B—C9B—C10B	−170.3 (4)
C23A—C24A—C25A—C20A	−0.3 (4)	C7B—C8B—C9B—N1B	−147.3 (6)
C21A—C20A—C25A—C24A	−0.5 (3)	C7B—C8B—C9B—C10B	31.5 (8)
C19A—C20A—C25A—C24A	177.6 (2)	N1B—C9B—C10B—C11B	37.3 (6)
N1X—N2B—C18B—S2B	−158.8 (4)	C8B—C9B—C10B—C11B	−141.6 (4)
N1B—N2B—C18B—S2B	178.0 (3)	C9B—C10B—C11B—C12B	−179.1 (4)
N1X—N2B—C18B—S1B	22.3 (5)	C10B—C11B—C12B—C13B	−162.8 (4)
N1B—N2B—C18B—S1B	−0.9 (4)	C10B—C11B—C12B—C17B	18.4 (6)
C19B—S1B—C18B—N2B	178.15 (18)	C17B—C12B—C13B—C14B	−1.1 (9)
C19B—S1B—C18B—S2B	−0.70 (19)	C11B—C12B—C13B—C14B	−180.0 (7)
C18B—S1B—C19B—C20B	−160.74 (18)	C12B—C13B—C14B—C15B	1.1 (18)
S1B—C19B—C20B—C21B	93.7 (3)	C13B—C14B—C15B—C16B	0 (3)
S1B—C19B—C20B—C25B	−85.7 (3)	C13B—C14B—C15B—O2B	175.8 (12)
C25B—C20B—C21B—C22B	−2.6 (4)	C27B—O2B—C15B—C16B	−4 (3)
C19B—C20B—C21B—C22B	177.9 (2)	C27B—O2B—C15B—C14B	−179.1 (12)
C20B—C21B—C22B—C23B	0.6 (4)	C14B—C15B—C16B—C17B	−2 (3)
C21B—C22B—C23B—C24B	1.9 (4)	O2B—C15B—C16B—C17B	−176.8 (14)
C22B—C23B—C24B—C25B	−2.3 (4)	C15B—C16B—C17B—C12B	1.9 (15)
C21B—C20B—C25B—C24B	2.2 (4)	C13B—C12B—C17B—C16B	−0.4 (8)
C19B—C20B—C25B—C24B	−178.4 (2)	C11B—C12B—C17B—C16B	178.4 (6)
C23B—C24B—C25B—C20B	0.3 (4)	C6X—C1X—C2X—C3X	−1 (5)
C9C—N1C—N2C—C18C	−179.3 (2)	C1X—C2X—C3X—C4X	1 (5)
C6C—C1C—C2C—C3C	0.6 (4)	C1X—C2X—C3X—O1B	−169 (3)
C26C—O1C—C3C—C2C	−20.9 (3)	C26B—O1B—C3X—C2X	−6 (3)
C26C—O1C—C3C—C4C	159.7 (2)	C26B—O1B—C3X—C4X	−176.5 (14)
C1C—C2C—C3C—O1C	−178.0 (2)	C2X—C3X—C4X—C5X	0 (4)
C1C—C2C—C3C—C4C	1.4 (4)	O1B—C3X—C4X—C5X	170.9 (18)
O1C—C3C—C4C—C5C	177.7 (2)	C3X—C4X—C5X—C6X	−1 (3)
C2C—C3C—C4C—C5C	−1.8 (4)	C2X—C1X—C6X—C5X	0 (4)
C3C—C4C—C5C—C6C	0.1 (4)	C2X—C1X—C6X—C7X	175 (3)
C2C—C1C—C6C—C5C	−2.2 (4)	C4X—C5X—C6X—C1X	1 (3)
C2C—C1C—C6C—C7C	175.4 (2)	C4X—C5X—C6X—C7X	−174.5 (18)
C4C—C5C—C6C—C1C	1.9 (4)	C1X—C6X—C7X—C8X	−163.8 (19)
C4C—C5C—C6C—C7C	−175.6 (2)	C5X—C6X—C7X—C8X	12 (3)
C1C—C6C—C7C—C8C	−161.5 (3)	C6X—C7X—C8X—C9X	174.1 (14)
C5C—C6C—C7C—C8C	16.0 (4)	C18B—N2B—N1X—C9X	−177.1 (6)
C6C—C7C—C8C—C9C	175.7 (2)	N2B—N1X—C9X—C8X	−2.0 (13)
N2C—N1C—C9C—C8C	−0.1 (3)	N2B—N1X—C9X—C10X	178.1 (7)
N2C—N1C—C9C—C10C	−177.19 (19)	C7X—C8X—C9X—N1X	−147.7 (13)
C7C—C8C—C9C—N1C	175.7 (2)	C7X—C8X—C9X—C10X	32.3 (16)
C7C—C8C—C9C—C10C	−7.4 (3)	N1X—C9X—C10X—C11X	−148.7 (8)
N1C—C9C—C10C—C11C	−26.9 (3)	C8X—C9X—C10X—C11X	31.4 (13)
C8C—C9C—C10C—C11C	155.8 (2)	C9X—C10X—C11X—C12X	173.8 (7)
C9C—C10C—C11C—C12C	177.1 (2)	C10X—C11X—C12X—C17X	−153.4 (8)
C10C—C11C—C12C—C13C	173.9 (2)	C10X—C11X—C12X—C13X	26.5 (12)
C10C—C11C—C12C—C17C	−7.4 (4)	C17X—C12X—C13X—C14X	3.0 (18)
C17C—C12C—C13C—C14C	1.7 (4)	C11X—C12X—C13X—C14X	−176.9 (14)
C11C—C12C—C13C—C14C	−179.6 (2)	C12X—C13X—C14X—C15X	0 (4)
C12C—C13C—C14C—C15C	−0.6 (4)	C27B—O2B—C15X—C16X	3 (6)
C27C—O2C—C15C—C14C	2.1 (4)	C27B—O2B—C15X—C14X	174 (3)
C27C—O2C—C15C—C16C	−177.4 (3)	C13X—C14X—C15X—O2B	−177 (2)
C13C—C14C—C15C—O2C	179.5 (3)	C13X—C14X—C15X—C16X	−4 (6)
C13C—C14C—C15C—C16C	−1.1 (4)	O2B—C15X—C16X—C17X	176 (4)
O2C—C15C—C16C—C17C	−178.8 (2)	C14X—C15X—C16X—C17X	5 (6)
C14C—C15C—C16C—C17C	1.6 (4)	C13X—C12X—C17X—C16X	−2.1 (15)
C15C—C16C—C17C—C12C	−0.5 (4)	C11X—C12X—C17X—C16X	177.8 (11)
C13C—C12C—C17C—C16C	−1.1 (3)	C15X—C16X—C17X—C12X	−2 (3)
C11C—C12C—C17C—C16C	−179.8 (2)	N2D—C18D—S1D—C19D	174.9 (3)
N1C—N2C—C18C—S2C	−172.08 (16)	S2D—C18D—S1D—C19D	−1.3 (3)
N1C—N2C—C18C—S1C	7.0 (3)	C18D—S1D—C19D—C20D	−162.9 (6)
C19C—S1C—C18C—N2C	176.62 (17)	S1D—C19D—C20D—C25D	70.4 (13)
C19C—S1C—C18C—S2C	−4.35 (19)	S1D—C19D—C20D—C21D	−108.5 (13)
C18C—S1C—C19C—C20C	−163.37 (17)	C25D—C20D—C21D—C22D	5 (2)
S1C—C19C—C20C—C25C	67.0 (3)	C19D—C20D—C21D—C22D	−176.0 (12)
S1C—C19C—C20C—C21C	−114.6 (2)	C20D—C21D—C22D—C23D	−3 (2)
C25C—C20C—C21C—C22C	0.0 (4)	C21D—C22D—C23D—C24D	0.0 (17)
C19C—C20C—C21C—C22C	−178.3 (2)	C22D—C23D—C24D—C25D	0.5 (15)
C20C—C21C—C22C—C23C	0.2 (4)	C21D—C20D—C25D—C24D	−4 (2)
C21C—C22C—C23C—C24C	−0.4 (4)	C19D—C20D—C25D—C24D	176.6 (11)
C22C—C23C—C24C—C25C	0.3 (4)	C23D—C24D—C25D—C20D	1.8 (19)
C23C—C24C—C25C—C20C	−0.1 (4)	C18D—S1Y—C19Y—C20Y	−130.3 (13)
C21C—C20C—C25C—C24C	−0.1 (4)	S1Y—C19Y—C20Y—C25Y	56 (3)
C19C—C20C—C25C—C24C	178.2 (2)	S1Y—C19Y—C20Y—C21Y	−135 (2)
C9D—N1D—N2D—C18D	179.8 (2)	C25Y—C20Y—C21Y—C22Y	−13 (4)
C6D—C1D—C2D—C3D	−1.0 (4)	C19Y—C20Y—C21Y—C22Y	177 (2)
C26D—O1D—C3D—C4D	175.9 (3)	C20Y—C21Y—C22Y—C23Y	7 (4)
C26D—O1D—C3D—C2D	−5.0 (4)	C21Y—C22Y—C23Y—C24Y	−1 (3)
C1D—C2D—C3D—O1D	−177.4 (2)	C22Y—C23Y—C24Y—C25Y	2 (3)
C1D—C2D—C3D—C4D	1.6 (4)	C21Y—C20Y—C25Y—C24Y	14 (4)
O1D—C3D—C4D—C5D	178.4 (2)	C19Y—C20Y—C25Y—C24Y	−177 (2)
C2D—C3D—C4D—C5D	−0.8 (4)	C23Y—C24Y—C25Y—C20Y	−8 (4)
C3D—C4D—C5D—C6D	−0.8 (4)		

### Hydrogen-bond geometry (Å, º)

*Cg*1, *Cg*2 and *Cg*3 are the centroids of the C12*B*–C17*B*, C12*C*–C17*C* and C1*B*–C6*B* rings, respectively.

**Table:**

*D*—H···*A*	*D*—H	H···*A*	*D*···*A*	*D*—H···*A*
C24*A*—H24*A*···O2*B*	0.93	2.57	3.262 (3)	131
C21*A*—H21*A*···O1*C*	0.93	2.40	3.249 (3)	152
C21*B*—H21*B*···O1*B*^i^	0.93	2.33	3.240 (4)	167
N2*C*—H3*N*2···S2*C*^ii^	0.91 (4)	2.53 (4)	3.435 (2)	179 (3)
C17*C*—H17*C*···O1*D*^iii^	0.93	2.53	3.381 (3)	152
N2*A*—H1*N*2···S2*A*^iv^	0.81 (3)	2.69 (3)	3.473 (2)	161 (2)
N2*B*—H2*N*2···S2*D*^v^	0.82 (3)	2.65 (3)	3.461 (2)	171 (3)
N2*D*—H4*N*2···S2*B*^v^	0.91 (4)	2.61 (4)	3.517 (2)	174 (3)
C14*C*—H14*C*···*Cg*1^iv^	0.93	2.80	3.558 (5)	139
C16*A*—H16*A*···*Cg*2^ii^	0.93	2.80	3.680 (2)	158
C2*D*—H2*DA*···*Cg*3	0.93	2.94	3.700 (5)	140

Symmetry codes: (i) −*x*, −*y*+2, −*z*; (ii) −*x*+2, −*y*, −*z*+1; (iii) −*x*+1, −*y*+1, −*z*+1; (iv) −*x*+1, −*y*, −*z*+1; (v) −*x*+1, −*y*+2, −*z*.
